# Joint Bayesian estimation of cell dependence and gene associations in spatially resolved transcriptomic data

**DOI:** 10.1038/s41598-024-60002-z

**Published:** 2024-04-25

**Authors:** Arhit Chakrabarti, Yang Ni, Bani K. Mallick

**Affiliations:** Department of Statistics, Texas A &M University, College Station, TX 77843 USA

**Keywords:** Single-cell, Spatial clustering, Spatially varying genes, Gene co-expression network, Statistics, Statistical methods

## Abstract

Recent technologies such as *spatial transcriptomics*, enable the measurement of gene expressions at the single-cell level along with the spatial locations of these cells in the tissue. Spatial clustering of the cells provides valuable insights into the understanding of the functional organization of the tissue. However, most such clustering methods involve some dimension reduction that leads to a loss of the inherent dependency structure among genes at any spatial location in the tissue. This destroys valuable insights of gene co-expression patterns apart from possibly impacting spatial clustering performance. In spatial transcriptomics, the matrix-variate gene expression data, along with spatial coordinates of the single cells, provides information on both gene expression dependencies and cell spatial dependencies through its row and column covariances. In this work, we propose a joint Bayesian approach to simultaneously estimate these gene and spatial cell correlations. These estimates provide data summaries for downstream analyses. We illustrate our method with simulations and analysis of several real spatial transcriptomic datasets. Our work elucidates gene co-expression networks as well as clear spatial clustering patterns of the cells. Furthermore, our analysis reveals that downstream spatial-differential analysis may aid in the discovery of unknown cell types from known marker genes.

## Introduction

Single-cell RNA-sequencing technologies have been used to create molecular profiles for individual cells, which provide valuable insights into the understanding of the composition of different cell types and their functions within a tissue. With newer technologies such as *spatial transcriptomics*, it is now possible to measure gene expressions at the single cell level along with the information of spatial locations of these cells in the tissue. Such technologies include the earlier fluorescence in situ hybridization (FISH) based approaches (e.g., seqFISH^1^ and MERFISH^2^), sequencing-based methods (e.g., 10x Visium^3^ and Slide-seq^4^), and the spatially-resolved transcript amplicon readout mapping (STARmap)^5^; see^6^ for a review of different spatial transcriptomic technologies. Spatial transcriptomic data bring new scientific questions and statistical challenges to its analysis and interpretation.

Spatial clustering is one of the most common exploratory analyses for spatial transcriptomic data. Spatial clustering aims to use spatial transcriptomic information to cluster cells in the tissue into multiple spatial clusters, thereby segmenting the entire tissue into multiple tissue structures or domains. This segmentation of the tissue structure may aid in the understanding of spatial and functional organization of the tissue. Common spatial clustering methods for spatial transcriptomic data include SpaGCN^7^, the hidden Markov random field model^8^, BayesSpace^9^, SpatialPCA^10^, and SC-MEB^11^. The majority of the popular spatial clustering methods, first involve a dimension reduction step on the expression matrix using some standard technique (e.g., PCA) followed by spatial clustering of the estimated low-dimensional embeddings. A more recent approach, DR.SC^12^ simultaneously achieves dimension reduction and spatial clustering, rather than performing them sequentially. However, although convenient for computational purposes, dimension reduction techniques often lead to the loss of the inherent dependency structure among genes (e.g., co-expression) at any spatial location in the tissue.

In many spatial transcriptomic studies (e.g., STARmap), the expression data are collected on a moderate number of genes for a large number of single cells along with their spatial information in the tissue. In such cases, it may be of interest to understand the association among the (sub)set of observed genes, along with the spatial clustering of the single cells. The existing spatial clustering methods perform dimension-reduction, either prior to clustering or simultaneously and hence, do not have provisions for understanding the genetic association. More concretely, the expression data observed for a set of *p* genes over a relatively large number *n* of single cells, constitute a matrix of expression data. The expression data are also accompanied with the $n\times d$ spatial co-ordinates of the single cells, where the dimension $d = 2 \text{ or } 3$ depends on the profiling method used. The matrix-variate spatial transcriptomic data provide information on both gene expression dependencies and cell spatial dependencies through the row and column covariances or correlations of the matrix-variate data.

Gaussian processes^13^ are commonly used to model spatial data, which typically involve the specification of spatial dependence in the form of a covariance matrix/kernel. Existing spatial covariance estimation methods ignore the dependency structure among the rows (genes in our case) of the matrix-variate data and often rely on a parametric assumption on the covariance kernel. The accuracy of covariance estimation may be sensitive to the specification of such kernels. SpatialDE^14^, SPARK^15^, and BOOST-GP^16^ adopt Gaussian processes with pre-specified parametric kernels to identify spatially varying genes. Moreover, genes are considered one-at-a-time to identify their spatial expression pattern. This possibly ignores interesting spatial expression patterns induced by co-expressing genes. In this paper, we propose a JOint BayeSian (JOBS) approach to simultaneously estimate the row and column covariances for the matrix-variate spatial transcriptomic data without fixing a parametric column covariance kernel or assuming the rows to be independent. Moreover, the proposed approach is computationally efficient for a large number of spatial locations (i.e., cells).

**Figure 1:**
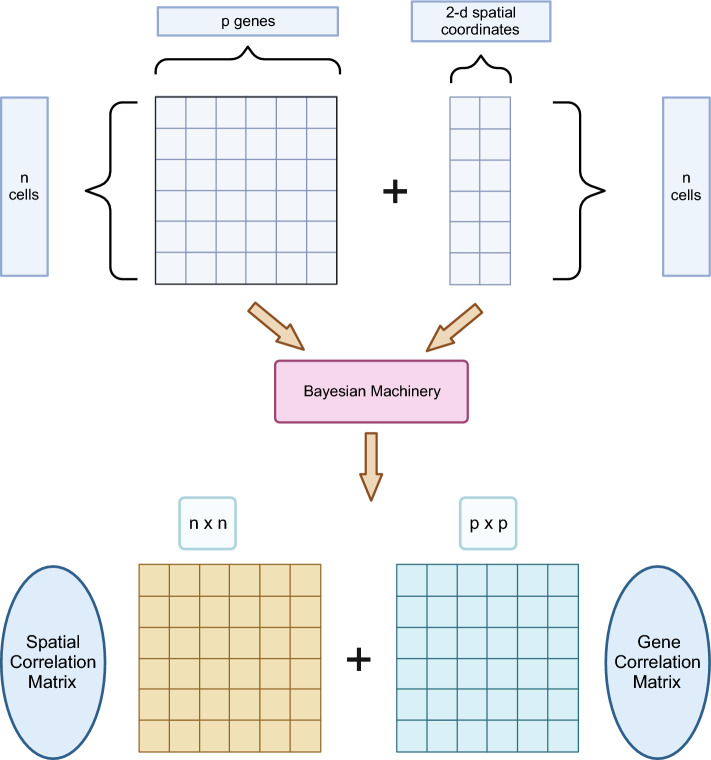
Illustration of our joint Bayesian methodology.

**Figure 2:**
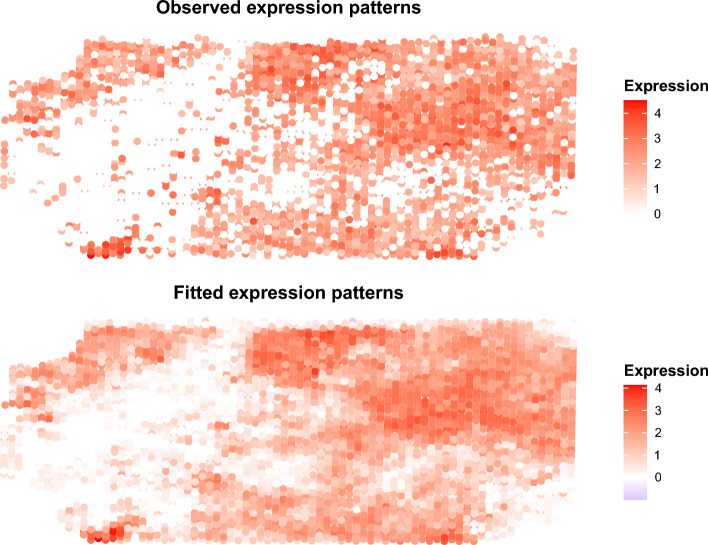
Observed and predicted spatial distribution of the gene “SCGB1D2” in the DLFPC dataset.

The proposed method (illustrated schematically in Fig. 1) takes as input the spatial gene expression matrix after standard log-normalization and the spatial coordinates of the single cells in the tissue. The JOBS output consists of the joint posterior estimates of both the row and column covariances for the matrix-variate spatial transcriptomic data. These posterior row and column correlation matrices are summaries of gene and cell dependencies, respectively. These outputs may be further processed and used for downstream analyses. For example, the estimated cell correlations (column correlation matrix in our case) may be used for jointly predicting the spatial distribution of a set of genes in the tissue whereas the estimated gene correlation matrix (corresponds to our row correlation matrix) may be used to reveal the gene co-expression patterns. As an illustration, the Figure 2 shows the observed and JOBS predicted spatial topology of the gene “SCGB1D2” in the dorsolateral prefrontal cortex (DLFPC) of the adult human brain^17^. These fitted gene expression data may be considered as a “de-noised” or smooth representation of the raw gene expression data and may be used for further downstream analyses. For example, the smoothed gene expression data can be used for spatial clustering of the cells in the tissue.

Existing methods of estimating gene co-expression network require the assumption that cell types (cluster labels) are either known or can be obtained via some existing spatial clustering method^18^. Our joint modeling approach circumvents this requirement by simultaneously providing the outputs for cell-type labelling and gene-network estimation. Our findings indicate that accurate estimation of the spatial correlation matrix is essential for achieving accurate cell clustering. Furthermore, we observed that misrepresenting gene correlations, such as assuming independence (uncorrelated), significantly impacts the estimation of spatial covariance. Overall, this article underscores the importance of precise spatial covariance estimation and highlights the detrimental effects of misrepresenting gene correlations. Additionally, our findings provide strong evidence supporting the superiority of our joint modeling approach in achieving improved cell clustering. Moreover, we extend our method for cells collected from multiple independent tissue samples through a Bayesian hierarchical model, which allows for the sharing of information across tissue samples even though the cell spatial locations could be different from tissue to tissue.

In this paper, we first performed detailed simulation experiments, comparing the performance of our proposed method with the existing spatial covariance estimation method in Section Simulations. We present an analysis of a real spatial transcriptomic dataset collected from the STARmap platform^5^ in Section STARmap data. In these studies, we demonstrated the effectiveness of our joint modeling approach, which incorporates both spatial and genomic level correlations, surpassing existing clustering methods. Additionally, we applied JOBS on two different spatial transcriptomics data obtained from the 10x Genomics Visium platform^17,19^. We discuss our findings and future directions for this work in Section Discussion. Section Discussion. provides a brief overview of our proposed joint Bayesian model for the case of a single-sample spatial transcriptomic data, and its extension to the case where we have multiple independent samples on a common gene set. The detailed description of our methodology, technical details of our hierarchical Bayesian model, and detailed simulation results can be found in the Supplementary.

## Results

### Simulations

The detailed simulation setup and its corresponding results are provided in the Supplementary Section D. We performed two sets of simulations to evaluate the performance of JOBS and compared it with a spatial covariance estimation approach ignoring the correlations among the genes (rows)^20^, called NPVecchia. We note that the estimated spatial correlation matrix can be used for spatial clustering of the cells in the tissue. Hence, its accurate estimation is essential to achieve precise spatial clustering. It is worthwhile to mention that existing spatial clustering methods that rely on PCA for dimension reduction consider these uncorrelated principal components for spatial clustering. Apart from destroying the inherent dependence between genes, we conjecture that using these uncorrelated principal components can lead to inefficient spatial clustering.

In our first set of simulations, we consider the case of a single sample of spatial transcriptomic data. We considered a wide range of simulation settings, with different choices of the true spatial covariance and gene covariance structures of the matrix-variate data. To monitor the accuracy of estimation of the spatial and gene correlation matrices, we compared the KL divergence (in log scale) and the relative Frobenius error. The technical definitions of KL divergence and relative Frobenius errors are provided in the Supplementary. We further, performed independent replications of our simulation experiments and reported the mean and standard deviation of the two metrics over the replications. From these replicated simulations, we found that in situations where the genes are correlated, the accuracy of estimation of both the gene and spatial correlation matrices is significantly higher for JOBS than under the NPVecchia. Thus, considering genes to be uncorrelated impacts the spatial correlation estimation, which in turn might have an effect on spatial clustering. We also note that as the number of spatial locations (single cells) increases, the accuracy of estimation of the gene correlations increases as can be seen from the corresponding decreasing relative estimation error. We refer the reader to Supplementary Section D.1 for the detailed results.

We extended JOBS to the case where there are multiple independent samples of spatial transcriptomic data. Specifically, we have independent samples of spatial transcriptomic data measured on the same set of genes over possible different spatial locations across samples. In our next set of simulations, we looked at the estimation accuracy of the covariance matrices in the presence of multiple independent samples of spatial transcriptomic data. For simplicity, we considered three independent samples of spatial data on the same set of genes (*p*) over possibly different spatial locations. The detailed simulation setup and results are presented in Supplementary Section D.2. We see that JOBS reports a significantly smaller estimation error of the covariance matrices than that from the NPVecchia. Moreover, the estimation error of the spatial covariance matrices decreases as the number of genes increases, whereas it shows an increasing trend for the competing method. Besides, as the number of spatial locations (single cells in our case) increases, the estimation error of the gene correlations decreases. Moreover, the estimation accuracy is higher than the case of a single sample of spatial transcriptomic data, which highlights the importance of having multiple samples.

Furthermore, we looked at the scalability of JOBS for increasing number of cells and features/genes through multiple independent replications. We note that JOBS scales nearly linearly with the number of cells. Additionally, the simulations show that the runtime is sub-linear with the number of features/genes. The detailed results can be found in the Supplementary Section D.3.

We note that although normalization is a standard pre-processing step for spatial transcriptomic data, the log-normalized matrix-variate data may be far from our assumed matrix normal distribution underneath our JOBS. We conducted sensitivity analysis for estimation accuracy, when the underlying data distribution is *non-normal*. In particular, we generated the data from a matrix-variate t distribution and looked at the efficiency of estimation for both one-sample and multi-sample case. As before, we considered a variety of number of spatial locations and varied the degrees of freedom of the corresponding matrix-variate t distribution. Clearly, from our results in Supplementary Section E, we see that the estimation performance under JOBS is better than that obtained from NPVecchia. It is worthwhile to note that under the mis-specified model, the estimation performance is sub-par in comparison to the case when the underlying data generating model is indeed matrix-variate normal. Also, an increase in the degrees of freedom of the matrix-variate t distribution shows an improved estimation performance, as such an increase in the degrees of freedom makes the data more “normal”. Furthermore, even under the mis-specified model, as the number of independent samples increases, the estimation errors of the row correlation matrices decreases, highlighting the importance of multiple samples of spatial transcriptomic data. The spatial correlation matrices also show lower estimation errors in comparison to the single-sample case, highlighting the benefits of our proposed hierarchical Bayesian model.

### STARmap data

We considered the STARmap (spatially-resolved transcript amplicon readout mapping) dataset^5^, which consists of data from four independent samples/mice. The experimental mice were dark housed for four days and then either exposed to light or kept in the dark for another one hour before obtaining measurements from the primary visual cortex. The data comprised of the expression of 160 genes with the number of cells varying between from 931 to 1167 for the four different samples. The spatial locations of these single cells in the tissue were also recorded. The STARmap study observed global induction of several known immediate-early genes in the primary visual cortex due to the light exposure as compared to the mice that were not exposed. This biologically interesting observation led us to focus our analysis on the two mice samples that were exposed to light. We refer to these as the “light” samples.

Genes that display spatial expression patterns in spatially resolved transcriptomic studies may help characterize the spatial transcriptomic landscape of complex tissues. Existing methods like BOOST-GP, SpatialDE, SPARK, and SPARK-X^21^ can identify the spatial expression patterns of genes, commonly referred to as Spatial Expression (SE) analysis. SE analysis can help choose the genes that show high spatial variations. However, these methods consider one gene at a time to estimate its spatial expression pattern. In many cases, there may exist co-expressing genes that induce interesting spatial distribution patterns. This motivated us to consider JOBS on the spatially varying genes for the two independent “light” samples. In particular, we selected the top 50 spatially variable genes using SPARK-X, implemented in the R package DR.SC^22^ for each of the two independent light samples and considered a common set of genes, which led to 33 spatially varying genes. As a standard pre-processing step for spatial transcriptomics data, we removed cells showing extreme expression of genes from each of the light samples. The data were subsequently log-normalized with a scaling factor equal to the median expression of total reads per cell, following the STARmap study protocol. Thus, the final analysis-ready dataset amounts to the log-normalized expression data for 33 spatially varying genes measured on 927 and 847 single-cells respectively for the two light samples.

**Figure 3:**
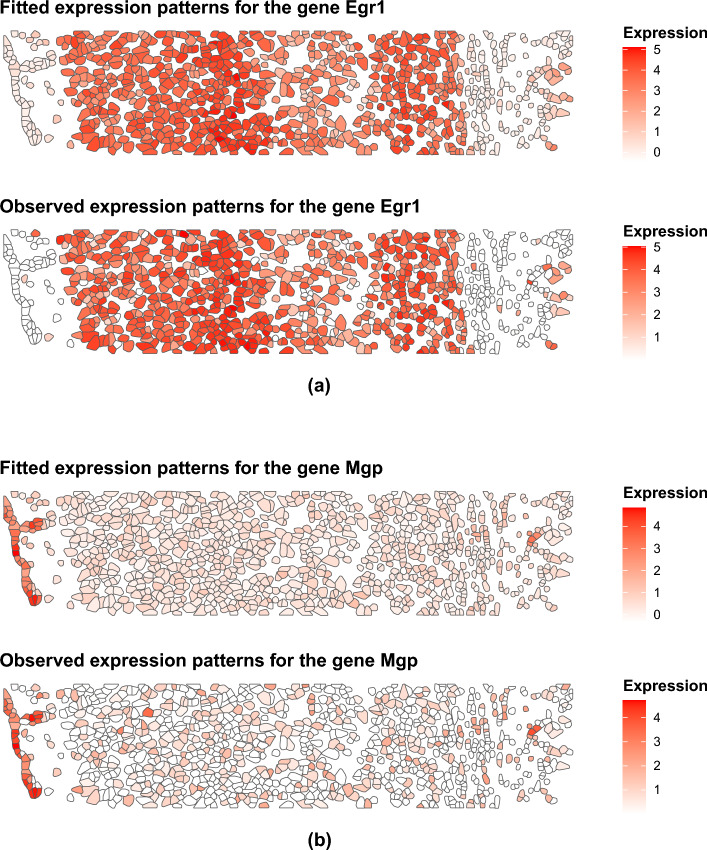
Smoothed and observed spatial expression patterns for two genes corresponding to one of the “light” samples.

**Figure 4:**
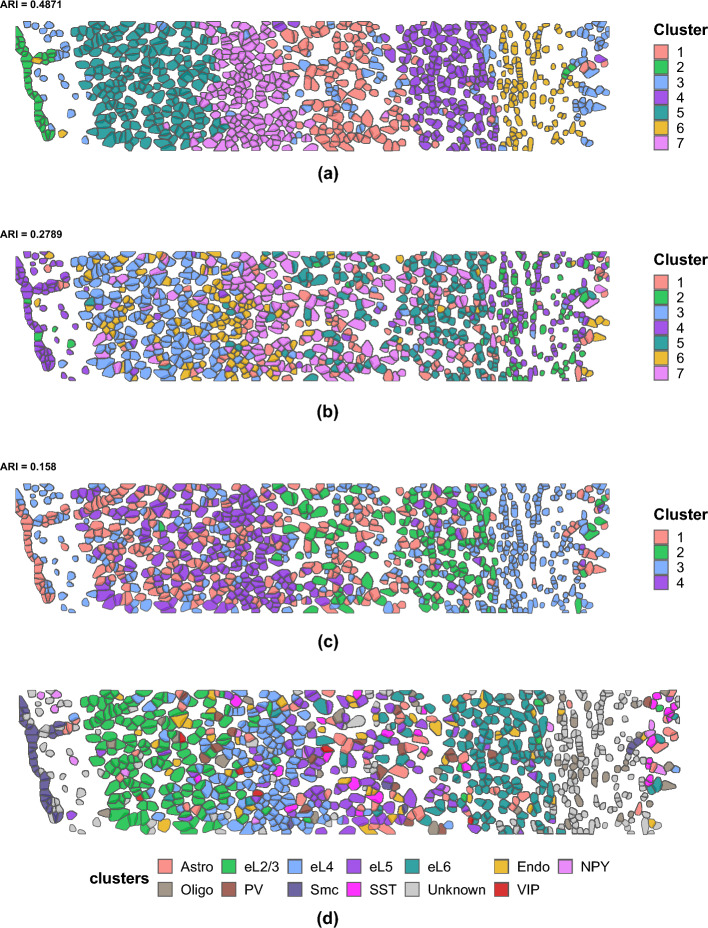
Spatial clustering from our proposed JOBS compared with the state of the art method as implemented by DR.SC R package and the BayesSpace for one of the light samples. The colors indicate the estimated clusters. ARI comparing the estimated cluster labels with the manually annotated cells (shown in (d)) is reported at the top of each panel.

We ran the proposed JOBS on the processed dataset, which provided us with the posterior estimate of a spatial correlation matrix for each sample and the shared gene correlation matrix across samples. We further post-processed these outputs to extract important features, particular to both the sample-specific and shared data. In particular, we obtained the smoothed spatial expression patterns, jointly for the 33 selected genes in each sample. The mean correlation and the mean squared error between the smoothed and observed gene expression values across the two samples were found to be 0.802 and 0.597 respectively. This indicated the high accuracy of the estimation of the spatial cell and gene covariance matrices for the STARmap data. Figure 3 shows the smoothed and observed spatial expression patterns for the genes “Egr1” and “Mgp”. Clearly, the smoothed expression patterns are highly aligned with the observed spatial distribution. We considered a Gaussian mixture model (GMM) on the smoothed gene expression data to obtain spatial clustering of the cells, choosing the optimal number of clusters using the Bayesian Information Criterion^23^. We compared the clustering results with two other well-known spatial clustering methods, namely *BayesSpace* and *DR.SC*. To objectively assess the clustering accuracy, we used the manually annotated cell types from the original STARmap study. Since excitatory cells formed a rich class of distinctly identified neurons, we focused our comparison on the subset of major cell types (eL2/3, eL4, eL5, and eL6) constituting excitatory cells. Figure 4 shows the clustering plot for one of the two light samples using the three methods. We looked at the Adjusted Rand Index^24^ to demonstrate clustering performance of the three competing methods, comparing the estimated cluster labels with the manually annotated cells types of excitatory cells. The corresponding plot with the true labels as obtained from the STARmap platform is shown in Fig. 4d (we looked at the subset of cell types eL2/3, eL4, eL5, and eL6). Furthermore, since *BayesSpace* requires the specification of the number of clusters, we ran the algorithm for multiple choices of the number of clusters and report the one with highest accuracy. Clearly, the clustering obtained from JOBS outperforms the other two methods in terms of clustering performance, specifically designed for spatial clustering. This highlights the importance of joint modelling of the gene and spatial correlations in spatial transcriptomic data, which possibly further enhances spatial clustering.

**Figure 5:**
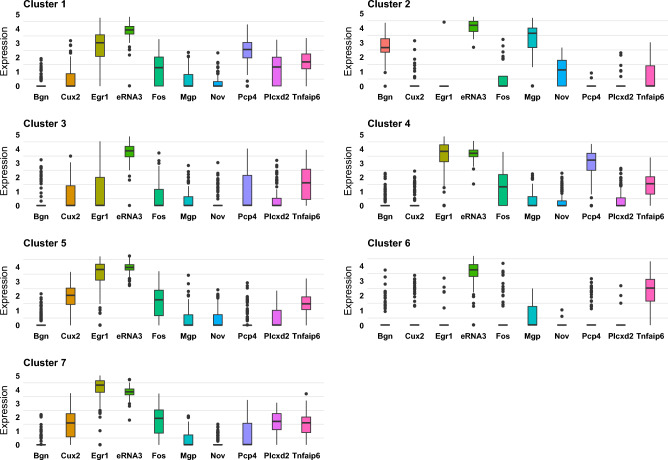
Boxplot of the expression of top ten spatially varying genes by cluster.

The boxplot of the expression of the top ten spatially varying genes (obtained from SPARK-X) across the different clusters estimated from JOBS in Figure 5 shows interesting distributional pattern. The gene “eRNA3” is seen to be almost uniformly distributed across the clusters, with a relatively high expression pattern. This uniform spatial distribution of the gene “Egr1” for the “light” sample is also validated by STARmap platform. Interestingly, the gene “Bgn” is only significantly expressed in cluster 2. “Bgn” encodes a member of the small leucine-rich proteoglycan family of proteins, which plays a role in bone growth, muscle development, and regeneration^25,26^. The STARmap platform validates most of these cells as smooth muscle cells, which constitute of involuntary, non-striated muscle as seen in Figure 4d. This possibly justifies the up-regulation of the gene “Bgn” in the cluster comprising of smooth muscle cells. Our analysis highlights that the joint modeling approach can aid in the identification of relevant marker genes by clusters.

**Figure 6:**
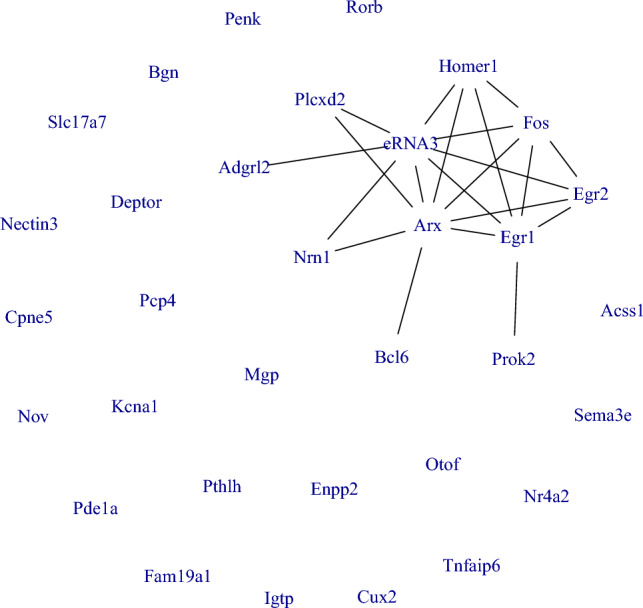
Estimated network between the spatially varying genes.

**Figure 7:**
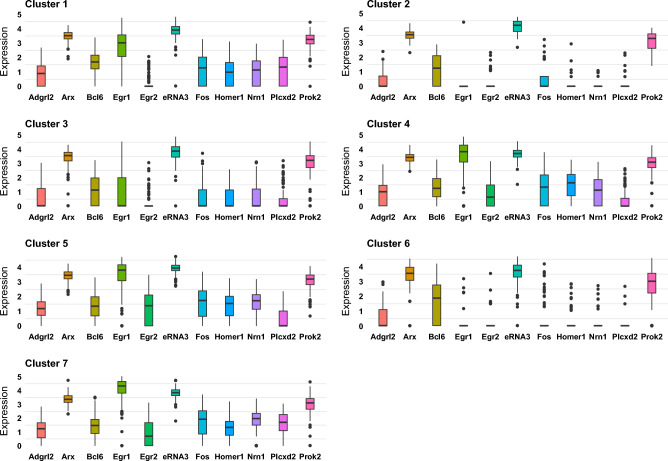
Boxplot of the expression of the co-expressed genes by cluster.

Using the posterior estimates of the spatial correlations, we de-correlated the data and, with graphical LASSO^27^, estimated the network among the selected genes, shown in Figure 6. The estimated network shows that the gene “eRNA3” and “Arx” are hub genes, showing association with multiple genes. Enhancers may be regarded as DNA sequences that regulate the gene expression networks. Enhancer RNAs (eRNAs), which are transcribed from enhancers in a tissue-specific manner, constitute an important class of non-coding RNAs with a multitude of functions involving gene expression regulation^28^. The STARmap study considered eRNAs 1 to 5 of the “Fos” gene and identified “eRNA3” as the most notable and consistent activity-regulated gene marker, which is also highlighted in our estimated network with “eRNA3” being a hub gene. Our estimated network captured this co-expression network between the genes “eRNA3” and “Fos”. Concurrently, the “Arx” gene provides instructions for producing a protein that regulates the activity of other genes^29,30^. Dickel et al.^31^ found that enhancers near “Arx” gene regulate its transcription in the mouse brain tissue. This possibly justifies the co-expression pattern between the genes “Arx” and “eRNA3” and the genes being hub genes. Furthermore, Figure 7 shows the expression of the co-expressed genes across the different clusters.

**Figure 8:**
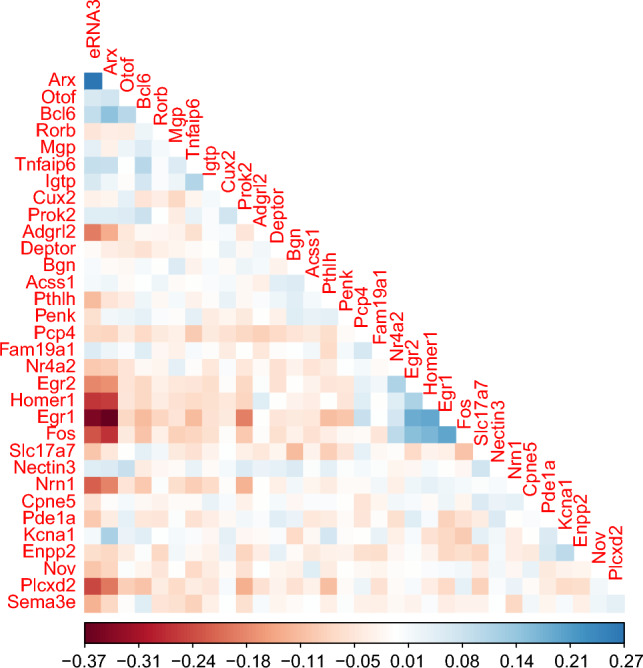
Heatmap of correlation between the spatially varying genes.

The posterior estimate of the row correlation matrix was used to visualize the correlations among the spatially varying genes in Figure 8. The plot shows positive correlation between “Arx” and “eRNA3”, which is again consistent with findings from existing literature. The plot shows high negative correlations of the gene “Egr1” with “Arx” and “Prok2”. Further, the estimated network in Fig. 6 shows that “Arx” is connected with “Prok2” through the gene “Egr1”. This possibly justifies the expression pattern of these genes in clusters 2 and 6, wherein up-regulation of “Arx” down-regulates expression of “Egr1”, which in turn up-regulates “Prok2”. The estimated correlations give support to the estimated network in Fig. 6 and the spatial distributional patterns in Fig. 5, revealing strong correlations among the co-expressed genes.

In addition to the STARmap data, we considered another two spatial transcriptomic datasets obtained from the 10x Genomics Visium platform. In particular, we considered the DLFPC dataset studied by^17^ and the human breast cancer dataset considered by^19^. The detailed description of the datasets and our analyses can be found in the Supplementary Section F. The proposed JOBS-based clustering produced a superior or similar spatial clustering compared to *DR.SC* and *BayesSpace*.

## Discussion

We have introduced a joint Bayesian method for the estimation of covariance matrices for matrix-variate spatial transcriptomic data wherein both the genes (rows) and cells (columns) of the matrix-variate data are correlated by the very design of the study. We have considered the case where we have multiple independent samples of the spatial transcriptomic data observed over a possibly different set of spatial locations but a common set of genes. We have illustrated the power of our method using both extensive simulations and real data where we made comparison with existing methods. The post-processed outputs from our method when used for spatial clustering shows improved clustering performance over existing methods. As opposed to existing methods, JOBS can be used to understand gene co-expression network as well as joint-differential analysis of these genes by clusters.

There are a few possible future directions for this work. First, it may be possible to consider spatial transcriptomic studies with large number of observed genes. The challenge is to define the joint distribution over the matrix-variate data, which along with the estimation of covariance matrices would allow for automatic selection of spatially varying genes from the entire gene set through some Bayesian variable selection criterion. It may be also possible to incorporate some Bayesian model-based clustering algorithm for the spatial clustering. Currently, we consider a Markov chain Monte Carlo (MCMC) algorithm to estimate the correlation matrices in our model. This possibly restricts the applicability of our method to large scale spatial transcriptomic datasets. However, it may be possible to consider a variational Bayes approach to estimating the JOBS model parameters, which would significantly speed up computational time.

## Methods

### Joint Covariance estimation for single-sample spatial transcriptomic data

In this section, we briefly present the proposed Bayesian methodology to jointly estimate the covariance matrices of a single-sample matrix-variate spatial transcriptomic data. The detailed methodology is presented in the Supplementary Section A.1. Consider an $p\times n$ matrix $\varvec{Y}$ of spatial transcriptomic data where *p* denotes the number of genes and *n* denotes the number of cells measured at the spatial locations $\varvec{s}_1, \dots , \varvec{s}_n$,

1 $$\begin{aligned} \varvec{Y} = \begin{pmatrix} y_1^{(1)} &{} \cdots &{} y_n^{(1)}\\ \vdots &{} \ddots &{} \vdots \\ y_1^{(p)} &{} \cdots &{} y_n^{(p)} \end{pmatrix} = \begin{pmatrix} |&{} &{} |\\ \varvec{y}_1&{} \cdots &{} \varvec{y}_n\\ |&{} &{} |\end{pmatrix}. \end{aligned}$$

Here $y_i^{(\ell )}$ is the expression of the $\ell$th gene in the *i*th cell at location $\varvec{s}_i$. We model $\varvec{Y}$ as a centered matrix-normal distribution,

2 $$\begin{aligned} \varvec{Y} \sim \mathscr{M}\mathscr{N}_{p, n}(0, \Lambda , \Sigma ), \end{aligned}$$

where $\Lambda$ and $\Sigma$ are the row and column covariance matrices. These correspond to the gene and spatial covariance matrices for the spatial transcriptomic data. We focus on problems where the number of spatial locations *n* is much larger than the number of genes *p*. To circumvent computational challenges we consider a sparse approximate Cholesky decomposition,

3 $$\begin{aligned} \Sigma ^{-1} = \text {U}\text {D}^{-1}\text {U}^\top , \end{aligned}$$

where $\text {D}=\text {diag}(d_1,\dots , d_n)$ is a diagonal matrix with positive entries $d_i >0$, and $\text {U}$ is a unit upper triangular matrix, i.e., an upper triangular matrix with diagonals equal to one.

We consider a maximin ordering^32,33^ of the spatial locations $\varvec{s}_1, \dots , \varvec{s}_n$, and accordingly the columns of $\varvec{Y}$. We further consider an ordered conditional independence assumption,

4 $$\begin{aligned} p(\varvec{y}_i\mid \varvec{y}_{1:i-1},\Lambda , \Sigma ) = p(\varvec{y}_i\mid \varvec{y}_{g_m(i)}, \Lambda , \Sigma ), \hspace{0.2cm} i=2, \dots , n, \end{aligned}$$

where $g_m(i) \subset \{1, \dots , i-1\}$ is an index vector consisting of the indices of the $\min (m, i-1)$ nearest neighbours to $\varvec{s}_i$ among those ordered previously. The ordered conditional independence in equation (4) implies that $\text {U}$ is sparse with at most *m* nonzero off-diagonal elements per column, thereby giving a sparse approximate modified Cholesky factorization of $\Sigma ^{-1}$.

#### Bayesian regression model framework

Under the maximin ordering constraint, $\text {U}$ and $\text {D}$ can be constructed directly by regressing each column $\varvec{y}_i$ of $\varvec{Y}$ on its predecessors^34^. Defining $\varvec{u}_i = \text {U}_{g_m(i),i}$ as the nonzero off-diagonal entries in the *i*th column of $\text {U}$, the model equation (2) can be written as a series of linear regression models:

5 $$\begin{aligned} p(\varvec{Y}\mid \Lambda , \Sigma )&= \prod _{i=1}^{n} p(\varvec{y}_i\mid \varvec{y}_{g_m(i)}, \Lambda , \Sigma ) = \prod _{i=1}^{n} \mathscr {N}_p(\varvec{y}_i\mid \varvec{X}_i\varvec{u}_i, d_i\Lambda ), \\ \end{aligned}$$

where the “design matrix” $\varvec{X}_i$ consists of the observations at the *m* neighboring locations of $\varvec{s}_i$, stored in the columns of $\varvec{Y}$ with indices $g_m(i)$. We let $m_i = |g_{m}(i)|$ to denote the cardinality of the index set $g_m(i)$. For efficient Bayesian inference of the model parameters, we assign conjugate shrinkage priors. For $i=1, \dots , n,$

6 $$\begin{aligned}&\varvec{u}_i\mid d_i\overset{ind}{\sim } & {} \ \ \mathscr {N}_{m_i}(\varvec{0}, d_i \varvec{V}_i), \\&d_i\overset{ind}{\sim } & {} \ \ \mathscr{I}\mathscr{G}(\alpha _i, \beta _i), \\&\Lambda\overset{ind}{\sim } & {} \ \ \mathscr{I}\mathscr{W}(\nu , \Psi ). \end{aligned}$$

Such conjugate priors lead to closed form updates for these parameters in our posterior sampling algorithm.

#### Parameterization and inference on the hyperparameters

We reparameterize the priors for $\varvec{u}_i$ and $d_i$ in Eq. (6) in terms of a much smaller number of hyperparameters. Inspired by the behavior of Matérn-type covariance functions, we introduce a three-dimensional vector of hyperparameters $\varvec{\theta } = (\theta _1, \theta _2, \theta _3)^\top$, where $\theta _1$ is related to the marginal variance, $\theta _2$ is related to the range, and $\theta _3$ is related to the smoothness. The motivation to reparameterize the priors stems from both empirical observations and theoretical results regarding the Cholesky factors in Eq. (3). To summarize, the hyperparameters of the priors in Eq.  (6) are related to $\varvec{\theta } = (\theta _1, \theta _2, \theta _3)^\top$ as follows. For $i = 1, \dots , n,$

7 $$\begin{aligned} \alpha _i&= 6 ,&\beta _i&= 5 \theta _1 (1 - \exp (-\theta _2(i)^{-\frac{1}{p}}), \\ \varvec{V}_i&= \text {Diag}(v_{i1}, \dots , v_{im_i}),&v_{ij}&= \frac{\exp (-\theta _3\,j)}{\theta _1(1 - \exp (-\theta _2(i)^{-\frac{1}{p}}))},\ \ j = 1, \dots , m_i. \end{aligned}$$

Here (*i*) is used to denote the nearest neighbor index. For a fully Bayesian inference, we further assume a flat prior for $\varvec{\theta }$. We adopt a Metropolis-Within-Blocked Gibbs approach to efficiently infer the model parameters. The details of our posterior inference algorithm are presented in the Supplementary Section A.1.4.

### Covariance estimation for spatial transcriptomic data with multiple independent samples

In many cases, we have independent samples of spatial transcriptomic data measured on the same set of genes. For example, the experiment may collect spatially resolved single-cell gene expression data for a set of genes of interest from a number of experimental units (e.g, different tissue samples). In this section, we extend the proposed method to such a case. Although we have independent samples of spatial transcriptomic data, the data may be observed over a different set of spatial locations for the different samples (e.g, the observed single cells have different spatial locations across the tissue samples). This problem is different from the traditional statistical setup of estimation using independent samples and brings in new statistical challenges. Under the assumption of the same underlying spatial field, we propose a Bayesian hierarchical model to allow for the borrowing of statistical strength across these independent samples.

Specifically, the data from the *r*th sample $\varvec{Y}_r$ is an $p \times n_r$ matrix, where $n_r$ denotes the number of single cells observed for the *r*th sample and *p* denotes the number of genes. The spatial locations of the single cells $\varvec{s}_{r1}, \dots , \varvec{s}_{rn_r}$ may not align for different samples $r,\, r= 1, \dots , R$. We consider the same maximin ordering of the spatial locations corresponding to each sample $\varvec{Y}_r$. Then each $\varvec{Y}_r$ is modeled independently as a centered matrix-normal distribution with a shared row covariance matrix but a sample-specific column covariance matrix,

$$\begin{aligned} \varvec{Y}_r \overset{ind}{\sim }\mathscr{M}\mathscr{N}_{p, n_r} (0, \Lambda , \Sigma _r), \ \ r = 1, \dots , R. \end{aligned}$$

Similarly as before, we take the modified Cholesky decomposition of the column precision matrix for each sample,

8 $$\begin{aligned} \Sigma _r^{-1} = \text {U}_r\text {D}_r^{-1}\text {U}_r^\top . \end{aligned}$$

Letting $\varvec{Y} = \{\varvec{Y}_1, \dots , \varvec{Y}_R\}$ denote the collection of all samples, we have a similar representation of the joint distribution of $\varvec{Y}$ in terms of series of linear regression models as Eq. (5),

9 $$\begin{aligned} p(\varvec{Y}\mid \Lambda , \{\Sigma _1,\dots , \Sigma _R\})&= \prod _{r=1}^{R}\prod _{i=1}^{n_r} p(\varvec{y}_{ri}\mid \varvec{y}_{rg_{r,m}(i)}, \Lambda , \Sigma _r) = \prod _{r=1}^{R}\prod _{i=1}^{n_r} \mathscr {N}_p(\varvec{y}_{ri}\mid \varvec{X}_{ri}\varvec{u}_{ri}, d_{ri}\Lambda ), \\ \end{aligned}$$

where the “design matrix” $\varvec{X}_{ri}$ of the *r*th sample consists of the observations at the *m* neighboring locations of $\varvec{s}_{ri}$, stored in the columns of $\varvec{Y}_r$ with indices $g_{r,m}(i)$. Similarly, $\varvec{u}_{ri} = \text {U}_{r, g_{r,m,i}(i)}$ is the nonzero off-diagonal entries in the *i*th column of $\text {U}_r$, and $d_{ri}$ is the *i*th diagonal element of the diagonal matrix $\text {D}_r$ in equation (8). Furthermore, we let $m_{ri} = |g_{r,m}(i)|$ to denote the cardinality of the index set $g_{r,m}(i)$. We assume independent priors that are conjugate to model Eq. (9), for $i=1, \dots , n_r,\ r=1, \dots , R,$

10 $$\begin{aligned}&\varvec{u}_{ri}\mid d_{ri}\overset{ind}{\sim } & {} \ \ \mathscr {N}_{m_{ri}}(\varvec{0}, d_{ri} \varvec{V}_{ri}),\\&d_{ri}\overset{ind}{\sim } & {} \ \ \mathscr{I}\mathscr{G}(\alpha _{ri}, \beta _{ri}),\\&\Lambda\overset{ind}{\sim } & {} \ \ \mathscr{I}\mathscr{W}(\nu , \Psi ). \end{aligned}$$

Similarly to Section Parameterization and inference on the hyperparameters, we reparameterize the priors for $\varvec{u}_{ri}$ and $d_{ri}$ in terms of a shared vector of hyperparameters $\varvec{\theta } = (\theta _1, \theta _2, \theta _3)^\top$. These hyperparameters are random (i.e., they have prior distributions) and *not* sample-dependent, and hence they allow for the sharing of the information across samples. This completes the specification of our Bayesian hierarchical model. We refer the reader to the Supplementary Section A.2 for a detailed description of the proposed model and posterior inference algorithm for multiple samples of spatial transcriptomic data.

### Supplementary Information

Supplementary Information.

## Data Availability

The STARmap data used in this work are publicly available from the website https://lce.biohpc.swmed.edu/star/index.html. The datasets from the 10x Visium are accessible on the 10x Genomics website at https://support.10xgenomics.com/spatial-gene-expression/datasets. The codes used for the analysis can be found in the repository https://github.com/Arhit-Chakrabarti/JOBS.
