# Supplementary material for: Joint Bayesian estimation of cell dependence and gene associations in spatially resolved transcriptomic data
Source: Sci Rep. 2024 Apr 25;14:9516. doi: 10.1038/s41598-024-60002-z (PMC11045727; doi:10.1038/s41598-024-60002-z)
Supplement: Supplementary file 1 — Supplementary Information. [file 41598_2024_60002_MOESM1_ESM.pdf]

Supplementary Materials for "Joint Bayesian Estimation of Cell Dependence and Gene Associations in Spatially Resolved Transcriptomic Data"

Arhit Chakrabarti[1,*], Yang Ni[1], and Bani K. Mallick[1]

[1]Texas A&M University, Department of Statistics, College Station, TX - 77843, USA
[*]arhit.chakrabarti@stat.tamu.edu

A Detailed Methodology

A.1 Joint Covariance estimation for single-sample spatial transcriptomic data

In this section, we give a detailed description of our proposed Bayesian methodology to jointly estimate the covariance matrices of a single-sample matrix-variate spatial transcriptomic data. Consider an $p \times n$ matrix $\boldsymbol{Y}$ of spatial transcriptomic data where p denotes the number of genes and n denotes the number of cells measured at the spatial locations $\boldsymbol{s}_1, \ldots, \boldsymbol{s}_n$,

$$\boldsymbol{Y} = \begin{pmatrix} y_1^{(1)} & \cdots & y_n^{(1)} \\ \vdots & \ddots & \vdots \\ y_1^{(p)} & \cdots & y_n^{(p)} \end{pmatrix} = \begin{pmatrix} | & & | \\ \boldsymbol{y}_1 & \cdots & \boldsymbol{y}_n \\ | & & | \end{pmatrix}. \tag{1}$$

Here $y_i^{(\ell)}$ is the expression of the ℓth gene in the ith cell at location $\boldsymbol{s}_i$. We model $\boldsymbol{Y}$ as a centered matrix-normal distribution,

$$\boldsymbol{Y} \sim \mathscr{MN}_{p,n}(0, \Lambda, \Sigma), \tag{2}$$

where Λ and Σ are the row and column covariance matrices. These correspond to the gene and spatial covariance matrices for the spatial transcriptomic data. We focus on problems where the number of spatial locations n is much larger than the number of genes p. In such problems, when the number n of columns of the matrix-variate data (the number of cells in our case) is large, it becomes difficult to store the dense matrix Σ in memory and the computation of Σ^{-1} becomes infeasible. Here, we propose a method to consider an approximate sparse Cholesky factorization for the column precision matrix Σ^{-1}. Rather than considering a standard Cholesky decomposition $LL^\top$ for Σ^{-1}, we use modified Cholesky decomposition, which has a better statistical interpretation [1]. In the modified Cholesky decomposition, Σ^{-1} is represented as

$$\Sigma^{-1} = \mathrm{U}\mathrm{D}^{-1}\mathrm{U}^\top, \tag{3}$$

where $\mathrm{D} = \mathrm{diag}(d_1, \ldots, d_n)$ is a diagonal matrix with positive entries $d_i > 0$, and U is a unit upper triangular matrix, i.e., an upper triangular matrix with diagonals equal to one.

There is a close connection between the modified Cholesky decomposition of a precision matrix and linear regression after appropriate ordering of the variables, which is the basis for more efficient algorithms. Hence, we order the spatial locations $\boldsymbol{s}_1, \ldots, \boldsymbol{s}_n$, and accordingly the columns of $\boldsymbol{Y}$ using the maximin ordering [2, 3]. The maximin ordering sequentially adds to the ordering, the location which maximizes the minimum distance from the locations already in the ordering. We further consider an ordered conditional independence assumption,

$$p(\boldsymbol{y}_i \mid \boldsymbol{y}_{1:i-1}, \Lambda, \Sigma) = p(\boldsymbol{y}_i \mid \boldsymbol{y}_{g_m(i)}, \Lambda, \Sigma), \quad i = 2, \ldots, n, \tag{4}$$

where $g_m(i) \subset \{1, \ldots, i-1\}$ is an index vector consisting of the indices of the $\min(m, i-1)$ nearest neighbours to $\boldsymbol{s}_i$ among those ordered previously. Note that equation (4) holds trivially for $m = n-1$. Many authors have demonstrated both numerically and theoretically that equation (4) holds (at least approximately) even for $m \ll n$ for many covariance functions in the context of Vecchia approximations of parametric covariance functions [2, 4, 5, 6, 7, 8, 9]. The ordered conditional independence in equation (4) implies that U is sparse with at most m nonzero off-diagonal elements per column, thereby giving a sparse approximate modified Cholesky factorization of Σ^{-1}.

Bayesian regression model framework

Under the maximin ordering constraint, U and D can be constructed directly by regressing each column $\boldsymbol{y}_i$ of $\boldsymbol{Y}$ on its predecessors [10]. That way, we can establish a connection between the modified Cholesky decomposition and linear regression as elucidated below. Defining $\boldsymbol{u}_i = \mathrm{U}_{g_m(i),i}$ as the nonzero off-diagonal entries in the ith column of U, the model equation (2) can be written as a series of linear regression models:

$$p(\boldsymbol{Y} \mid \Lambda, \Sigma) = \prod_{i=1}^{n} p(\boldsymbol{y}_i \mid \boldsymbol{y}_{g_m(i)}, \Lambda, \Sigma) = \prod_{i=1}^{n} \mathcal{N}_p(\boldsymbol{y}_i \mid \boldsymbol{X}_i \boldsymbol{u}_i, d_i \Lambda), \tag{5}$$

where the "design matrix" $\boldsymbol{X}_i$ consists of the observations at the m neighboring locations of $\boldsymbol{s}_i$, stored in the columns of $\boldsymbol{Y}$ with indices $g_m(i)$, i.e. $\boldsymbol{X}_i$ is an $p \times m$ matrix with the ℓth row $-\boldsymbol{y}_{g_m(i)}^{(\ell)\top}$. Note that under this notation $\mathbf{X}_1 = \mathbf{0}_{p\times 1}$. We let $m_i = |g_{m(i)}|$ to denote the cardinality of the index set $g_m(i)$. For efficient Bayesian inference of the model parameters, we assign conjugate shrinkage priors. For $i = 1, \dots, n$,

$$\begin{aligned} \boldsymbol{u}_i \mid d_i &\overset{ind}{\sim} \mathcal{N}_{m_i}(\mathbf{0}, d_i \boldsymbol{V}_i), \\ d_i &\overset{ind}{\sim} \mathcal{IG}(\alpha_i, \beta_i), \\ \Lambda &\overset{ind}{\sim} \mathcal{IW}(\nu, \Psi). \end{aligned} \tag{6}$$

If the number p of observed genes is moderate, an Inverse-Wishart ($\mathcal{IW}$) prior on the row covariance matrix Λ leads to closed form expressions for the full conditional distributions. When p is large, Bayesian latent factor models could be used instead [11, 12, 13].

Full conditional distributions

The full-conditional distributions of the model parameters $\boldsymbol{u} = \{\boldsymbol{u}_1, \dots, \boldsymbol{u}_n\}$, $\boldsymbol{d} = \{d_1, \dots, d_n\}$, and Λ are straightforward to derive. Specifically, the full-conditional distribution of $(\boldsymbol{u}, \boldsymbol{d})$ is given by,

$$p(\boldsymbol{u}, \boldsymbol{d} \mid \boldsymbol{Y}, \Lambda) \equiv \prod_{i=1}^{n} \mathcal{N}_{m_i}\left(\boldsymbol{u}_i \mid \boldsymbol{G}_i^{-1}\boldsymbol{H}_i, d_i \boldsymbol{G}_i^{-1}\right) \mathcal{IG}(d_i \mid \tilde{\alpha}_i, \tilde{\beta}_i), \tag{7}$$

where

$$\begin{aligned} \tilde{\alpha}_i &= \alpha_i + \frac{p}{2}, \\ \tilde{\beta}_i &= \beta_i + \frac{1}{2}\boldsymbol{y}_i^\top \left(\Lambda^{-1} - \Lambda^{-1}\boldsymbol{X}_i\boldsymbol{G}_i^{-1}\boldsymbol{X}_i^\top\Lambda^{-1}\right)\boldsymbol{y}_i, \\ \boldsymbol{G}_i^{-1} &= \left(\boldsymbol{V}_i^{-1} + \boldsymbol{X}_i^\top\Lambda^{-1}\boldsymbol{X}_i\right)^{-1}, \\ \boldsymbol{H}_i &= \boldsymbol{X}_i^\top\Lambda^{-1}\boldsymbol{y}_i. \end{aligned} \tag{8}$$

The full conditional distribution of Λ is given by,

$$p(\Lambda \mid \boldsymbol{Y}, \boldsymbol{u}, \boldsymbol{d}) \equiv \mathcal{IW}\left(\Psi + \sum_{i=1}^{n} \frac{(\boldsymbol{y}_i - \boldsymbol{X}_i\boldsymbol{u}_i)(\boldsymbol{y}_i - \boldsymbol{X}_i\boldsymbol{u}_i)^\top}{d_i}, n + \nu\right). \tag{9}$$

Because equation (7) and equation (9) are in closed form, Gibbs sampling is straightforward.

Parameterization and inference on the hyperparameters

We reparameterize the priors for $\boldsymbol{u}_i$ and d_i in equation (6) in terms of a much smaller number of hyperparameters. Inspired by the behavior of Matérn-type covariance functions, we introduce a three-dimensional vector of hyperparameters $\boldsymbol{\theta} = (\theta_1, \theta_2, \theta_3)^\top$, where θ_1 is related to the marginal variance, θ_2 is related to the range, and θ_3 is related to the smoothness. The motivation to reparameterize the priors stems from both empirical observations and theoretical results regarding the Cholesky factors in equation (3).

We first consider the prior for d_i in equation (6). Considering an exponential covariance kernel with marginal variance θ_1 and range $2/\theta_2$, the (i, j)th entry of the spatial covariance matrix, $\Sigma_{ij} = \theta_1 \exp(-\theta_2\|\boldsymbol{s}_i - \boldsymbol{s}_j\|/2)$. Assuming $m = 1$, we have,

$$\begin{aligned} \mathrm{Var}(\boldsymbol{y}_i \mid \boldsymbol{y}_{g_m(i)}, \Lambda) &= d_i\Lambda \\ &= \theta_1(1 - \exp(-\theta_2\|\boldsymbol{s}_i - \boldsymbol{s}_g\|))\Lambda \quad (10) \\ &\approx \theta_1(1 - \exp(-\theta_2(i)^{-\frac{1}{p}})\Lambda, \quad (11) \end{aligned}$$

where $g = g_1(i)$. The functional form in equation (10) holds exactly for the exponential covariance kernel with $m = 1$ and holds at least approximately for Mateŕn covariance kernels in two dimensions with $m = n-1$. The approximation in equation (11) follows since under a maximin ordering of the spatial locations, the distance $\|\boldsymbol{s}_i - \boldsymbol{s}_g\|$ between the location $\boldsymbol{s}_i$ and its nearest previously ordered neighbor decreases roughly as $(i)^{-1/p}$ for a regular grid on a unit hypercube $[0,1]^p$. The prior mean and variance of $d_i\Lambda$ conditional on Λ is given by,

$$\mathbb{E}(d_i\Lambda) = \frac{\beta_i}{\alpha_i - 1}\Lambda, \tag{12}$$

$$\mathrm{Var}(d_i\Lambda) = \frac{\beta_i^2}{(\alpha_i-1)^2(\alpha_i-2)}\Lambda^2. \tag{13}$$

With the motivation of getting a prior for d_i that shrinks toward equation (11), we set the prior mean equation (12) to be equal to the functional form in equation (11). Thus, for $i = 1,\ldots,n$,

$$\begin{aligned} &\frac{\beta_i}{\alpha_i-1}\Lambda = \theta_1(1-\exp(-\theta_2(i)^{-\frac{1}{p}})\Lambda \\ \Rightarrow &\frac{\beta_i}{\alpha_i-1} = \theta_1 f_{\theta_2}(i), \end{aligned} \tag{14}$$

where $f_{\theta_2}(i) = (1-\exp(-\theta_2(i)^{-\frac{1}{p}})$. As the empirically observed variance of d_i decreases with the index i as well, following this reasoning, we set the prior standard deviation of $d_i\Lambda$ (obtained from equation (13)) to be half the prior mean in equation (12). Therefore, for $i = 1,\ldots,n$,

$$\begin{aligned} &\frac{\beta_i}{(\alpha_i-1)\sqrt{\alpha_i-2}}\Lambda = \frac{\beta_i}{2(\alpha_i-1)}\Lambda \\ \Rightarrow &\frac{\beta_i}{(\alpha_i-1)\sqrt{\alpha_i-2}} = \frac{\beta_i}{2(\alpha_i-1)}. \end{aligned} \tag{15}$$

Solving for α_i and β_i from the equations equation (14) and equation (15) yields, for $i = 1,\ldots,n$,

$$\begin{aligned} &\alpha_i = 6 \\ &\beta_i = 5\theta_1 f_{\theta_2}(i). \end{aligned} \tag{16}$$

Recent results based on elliptic boundary-value problems imply that the Cholesky entry $(\boldsymbol{u}_i)_j$, corresponding to the jth nearest neighbor decays exponentially as a function of j for Mateŕn covariance functions whose spectral densities are the reciprocal of a polynomial (ignoring edge effects) [3]. With the motivation to capture this exponential decay for the Cholesky entries, the functional form for the entries v_{ij} of the diagonal matrix $\boldsymbol{V}_i$ in equation (6) are given by,

$$\boldsymbol{V}_i = \mathrm{Diag}(v_{i1},\ldots,v_{im_i}), \quad \text{where } v_{ij} = \frac{\exp(-\theta_3 j)}{\theta_1 f_{\theta_2}(i)}, \quad j = 1,\ldots,m_i, \; i = 1,\ldots,n. \tag{17}$$

To summarize, the hyperparameters of the priors in equation (6) are related to $\boldsymbol{\theta} = (\theta_1,\theta_2,\theta_3)^\top$ as follows. For $i = 1,\ldots,n$,

$$\begin{aligned} &\alpha_i = 6, && \beta_i = 5\theta_1(1-\exp(-\theta_2(i)^{-\frac{1}{p}}), \\ &\boldsymbol{V}_i = \mathrm{Diag}(v_{i1},\ldots,v_{im_i}), && v_{ij} = \frac{\exp(-\theta_3 j)}{\theta_1 f_{\theta_2}(i)}, \quad j = 1,\ldots,m_i. \end{aligned} \tag{18}$$

Here (i) is used to denote index. We note that all components of $\boldsymbol{\theta}$ are assumed to be positive and so we perform all inference on the logarithmic scale. We next discuss how to infer the hyperparameter $\boldsymbol{\theta}$ based on the data $\boldsymbol{Y}$. The key component for learning about the hyperparameter $\boldsymbol{\theta} = (\theta_1,\theta_2,\theta_3)^\top$ is the marginal or integrated likelihood,

$$p(\boldsymbol{Y} \mid \Lambda, \boldsymbol{\theta}) = \prod_{i=1}^{n} \int_{d_i}\int_{\boldsymbol{u}_i} \mathcal{N}_p(\boldsymbol{y}_i \mid \boldsymbol{X}_i\boldsymbol{u}_i, d_i\Lambda)\, \mathcal{N}_{m_i}(\boldsymbol{u}_i \mid \boldsymbol{0}, d_i\boldsymbol{V}_i)\, \mathcal{IG}(d_i \mid \alpha_i,\beta_i)\, \mathrm{d}d_i \mathrm{d}\boldsymbol{u}_i. \tag{19}$$

Standard calculations on multivariate normal distribution yield,

$$p(\boldsymbol{Y} \mid \Lambda, \boldsymbol{\theta}) \propto \prod_{i=1}^{n} \frac{|\boldsymbol{G}_i^{-1}|^{\frac{1}{2}}}{|\boldsymbol{V}_i|^{\frac{1}{2}}} \frac{\beta_i^{\alpha_i}}{\tilde{\beta}_i^{\tilde{\alpha}_i}} \frac{\Gamma(\tilde{\alpha}_i)}{\Gamma(\alpha_i)},$$

where the prior parameters $\alpha_i, \beta_i, \boldsymbol{V}_i$ are given in equation (6), and the parameters $\tilde{\alpha}_i, \tilde{\beta}_i, \boldsymbol{G}_i^{-1}$ are given in equation (8). The functional dependencies of the prior parameters $\alpha_i, \beta_i, \boldsymbol{V}_i$ on the hyperparameter $\boldsymbol{\theta}$ are specified by equation (18). For a fully Bayesian inference, we assume a flat prior for $\boldsymbol{\theta}$, and the marginal posterior distribution of $\boldsymbol{\theta}$ is given by,

$$\begin{aligned} p(\boldsymbol{\theta} \mid \Lambda, \boldsymbol{Y}) &\propto p(\boldsymbol{Y} \mid \Lambda, \boldsymbol{\theta}) \\ &\propto \prod_{i=1}^{n} \frac{|\boldsymbol{G}_i^{-1}|^{\frac{1}{2}}}{|\boldsymbol{V}_i|^{\frac{1}{2}}} \frac{\beta_i^{\alpha_i}}{\tilde{\beta}_i^{\tilde{\alpha}_i}} \frac{\Gamma(\tilde{\alpha}_i)}{\Gamma(\alpha_i)}. \end{aligned} \tag{20}$$

Blocked Gibbs sampling algorithm

From the full conditional distributions equation (7), equation (9), and the marginal posterior distribution equation (20), the blocked Gibbs sampling is straightforward. The algorithm iterates by sampling from the conditional distributions,

$$\begin{aligned} p(\boldsymbol{\theta} \mid \Lambda, \boldsymbol{Y}) &\propto \prod_{i=1}^{n} \frac{|\boldsymbol{G}_i^{-1}|^{\frac{1}{2}}}{|\boldsymbol{V}_i|^{\frac{1}{2}}} \frac{\beta_i^{\alpha_i}}{\tilde{\beta}_i^{\tilde{\alpha}_i}} \frac{\Gamma(\tilde{\alpha}_i)}{\Gamma(\alpha_i)}, \\ p(\boldsymbol{u}, \boldsymbol{d} \mid \boldsymbol{Y}, \Lambda, \boldsymbol{\theta}) &\propto \prod_{i=1}^{n} \mathcal{N}_{m_i}\left(\boldsymbol{u}_i \mid \hat{\boldsymbol{\mu}}_i, \hat{\boldsymbol{V}}_i\right) \mathcal{IG}(d_i \mid \tilde{\alpha}_i, \tilde{\beta}_i), \\ p(\Lambda \mid \boldsymbol{Y}, \boldsymbol{u}, \boldsymbol{d}, \boldsymbol{\theta}) &\propto \mathcal{IW}\left(\hat{\Psi}, n + \nu\right), \end{aligned} \tag{21}$$

where,

$$\hat{\boldsymbol{\mu}}_i = \boldsymbol{G}_i^{-1}\boldsymbol{H}_i, \qquad \hat{\boldsymbol{V}}_i = d_i \boldsymbol{G}_i^{-1}, \qquad \hat{\Psi} = \Psi + \sum_{i=1}^{n} \frac{(\boldsymbol{y}_i - \boldsymbol{X}_i \boldsymbol{u}_i)(\boldsymbol{y}_i - \boldsymbol{X}_i \boldsymbol{u}_i)^\top}{d_i}.$$

A.2 Covariance estimation for spatial transcriptomic data with multiple independent samples

We have extended our proposed method to the case when there are multiple independent samples of spatial transcriptomic data. In this section, we provide a detailed description of the methodology for the multi-sample case. Recall that in many cases, we have independent samples of spatial transcriptomic data measured on the same set of genes. For example, the experiment may collect spatially resolved single-cell gene expression data for a set of genes of interest from a number of experimental units (e.g, different tissue samples). Although we have independent samples of spatial transcriptomic data, the data may be observed over a different set of spatial locations for the different samples (e.g, the observed single cells have different spatial locations across the tissue samples). This problem is different from the traditional statistical setup of estimation using independent samples and brings in new statistical challenges. Under the assumption of the same underlying spatial field, we propose a Bayesian hierarchical model to allow for the borrowing of statistical strength across these independent samples.

Specifically, the data from the rth sample $\boldsymbol{Y}_r$ is an $p \times n_r$ matrix, where n_r denotes the number of single cells observed for the rth sample and p denotes the number of genes. The spatial locations of the single cells $\boldsymbol{s}_{r1}, \ldots, \boldsymbol{s}_{r,n_r}$ may not align for different samples r, $r = 1, \ldots, R$. We consider the same maximin ordering of the spatial locations corresponding to each sample $\boldsymbol{Y}_r$. Then each $\boldsymbol{Y}_r$ is modelled independently as a centered matrix-normal distribution with a shared row covariance matrix but a sample-specific column covariance matrix,

$$\boldsymbol{Y}_r \overset{ind}{\sim} \mathcal{MN}_{p,n_r}(0, \Lambda, \Sigma_r), \quad r = 1, \ldots, R.$$

Similarly as before, we take the modified Cholesky decomposition of the column precision matrix for each sample,

$$\Sigma_r^{-1} = \mathrm{U}_r \mathrm{D}_r^{-1} \mathrm{U}_r^\top. \tag{22}$$

Letting $\boldsymbol{Y} = \{\boldsymbol{Y}_1, \ldots, \boldsymbol{Y}_R\}$ denote the collection of all samples, we have a similar representation of the joint distribution of $\boldsymbol{Y}$ in terms of series of linear regression models as equation (5),

$$p(\boldsymbol{Y} \mid \Lambda, \{\Sigma_1, \ldots, \Sigma_R\}) = \prod_{r=1}^{R} \prod_{i=1}^{n_r} p(\boldsymbol{y}_{ri} \mid \boldsymbol{y}_{rg_{r,m(i)}}, \Lambda, \Sigma_r) = \prod_{r=1}^{R} \prod_{i=1}^{n_r} \mathcal{N}_p(\boldsymbol{y}_{ri} \mid \boldsymbol{X}_{ri}\boldsymbol{u}_{ri}, d_{ri}\Lambda), \tag{23}$$

where the "design matrix" $\boldsymbol{X}_{ri}$ of the rth sample consists of the observations at the m neighboring locations of $\boldsymbol{s}_{ri}$, stored in the columns of $\boldsymbol{Y}_r$ with indices $g_{r,m(i)}$. Similarly, $\boldsymbol{u}_{ri} = \mathrm{U}_{r,g_{r,m(i)},i}$ is the nonzero off-diagonal entries in the ith column of U_r, and d_{ri} is

the ith diagonal element of the diagonal matrix D_r in equation (22). Furthermore, we let $m_{ri} = |g_{r,m(i)}|$ to denote the cardinality of the index set $g_{r,m(i)}$. We assume independent priors that are conjugate to model equation (23), for $i = 1,\dots,n_r,\ r = 1,\dots,R$,

$$\begin{aligned}
\boldsymbol{u}_{ri} \mid d_{ri} &\overset{ind}{\sim} \mathcal{N}_{m_{ri}}(\mathbf{0}, d_{ri}\boldsymbol{V}_{ri}),\\
d_{ri} &\overset{ind}{\sim} \mathcal{IG}(\alpha_{ri}, \beta_{ri}),\\
\Lambda &\overset{ind}{\sim} \mathcal{IW}(\nu, \Psi).
\end{aligned} \tag{24}$$

Similarly to Section A.1, we reparameterize the priors for $\boldsymbol{u}_{ri}$ and d_{ri} in terms of a shared vector of hyperparameters $\boldsymbol{\theta} = (\theta_1, \theta_2, \theta_3)^\top$. Note that these hyperparameters are random (i.e., they have prior distributions) and *not* sample-dependent, and hence they allow for the sharing of the information across samples. The blocked Gibbs sampling algorithm for the multi-sample case follows as an immediate extension to the corresponding single-sample algorithm. Letting $\boldsymbol{u} = \{\boldsymbol{u}_{ri}, i = 1,\dots,n_r,\ r = 1,\dots,R\}$ and $\boldsymbol{d} = \{d_{ri}, i = 1,\dots,n_r,\ r = 1,\dots,R\}$, the algorithm iterates by sampling,

$$\begin{aligned}
p(\boldsymbol{\theta} \mid \Lambda, \boldsymbol{Y}) &\propto \prod_{r=1}^{R}\prod_{i=1}^{n_r} \frac{|\boldsymbol{G}_{ri}^{-1}|^{\frac{1}{2}}}{|\boldsymbol{V}_{ri}|^{\frac{1}{2}}} \frac{\beta_{ri}^{\alpha_{ri}}}{\tilde{\beta}_{ri}^{\tilde{\alpha}_{ri}}} \frac{\Gamma(\tilde{\alpha}_{ri})}{\Gamma(\alpha_{ri})},\\
p(\boldsymbol{u}, \boldsymbol{d} \mid \boldsymbol{Y}, \Lambda, \boldsymbol{\theta}) &\propto \prod_{r=1}^{R}\prod_{i=1}^{n_r} \left\{ \mathcal{N}_{m_{ri}}\left(\boldsymbol{u}_{ri} \mid \hat{\boldsymbol{\mu}}_{ri}, \hat{\boldsymbol{V}}_{ri}\right) \mathcal{IG}(d_{ri} \mid \tilde{\alpha}_{ri}, \tilde{\beta}_{ri}) \right\},\\
p(\Lambda \mid \boldsymbol{Y}, \boldsymbol{u}, \boldsymbol{d}, \boldsymbol{\theta}) &\propto \mathcal{IW}\left(\hat{\Psi}, \sum_{r=1}^{R} n_r + \nu\right),
\end{aligned} \tag{25}$$

where, for $i = 1,\dots,n_r$, $r = 1,\dots,R$,

$$\begin{aligned}
&\alpha_{ri} = 6, && \tilde{\alpha}_{ri} = \alpha_{ri} + \frac{p}{2},\\
&\beta_{ri} = 5\theta_1(1 - \exp(-\theta_2 (i)^{-\frac{1}{p}}), &&\\
&\tilde{\beta}_{ri} = \beta_{ri} + \frac{1}{2}\boldsymbol{y}_{ri}^\top \left(\Lambda^{-1} - \Lambda^{-1}\boldsymbol{X}_{ri}\boldsymbol{G}_{ri}^{-1}\boldsymbol{X}_{ri}^\top\Lambda^{-1}\right)\boldsymbol{y}_{ri}, &&\\
&\boldsymbol{G}_{ri}^{-1} = \left(\boldsymbol{V}_{ri}^{-1} + \boldsymbol{X}_{ri}^\top\Lambda^{-1}\boldsymbol{X}_{ri}\right)^{-1}, && \boldsymbol{H}_{ri} = \boldsymbol{X}_{ri}^\top\Lambda^{-1}\boldsymbol{y}_{ri},\\
&\hat{\boldsymbol{\mu}}_{ri} = \boldsymbol{G}_{ri}^{-1}\boldsymbol{H}_{ri}, && \hat{\boldsymbol{V}}_{ri} = d_{ri}\boldsymbol{G}_{ri}^{-1},\\
&\hat{\Psi} = \Psi + \sum_{r=1}^{R}\sum_{i=1}^{n_r} \frac{(\boldsymbol{y}_{ri} - \boldsymbol{X}_{ri}\boldsymbol{u}_{ri})(\boldsymbol{y}_{ri} - \boldsymbol{X}_{ri}\boldsymbol{u}_{ri})^\top}{d_{ri}}, &&\\
&\boldsymbol{V}_{ri} = \mathrm{Diag}(v_{ri1}, \dots, v_{rim_{ri}}), && v_{rij} = \frac{\exp(-\theta_3 j)}{\theta_1(1 - \exp(-\theta_2 (i)^{-\frac{1}{p}})}.
\end{aligned} \tag{26}$$

In all our analyses, we fix the maximum number of neighbors $m = 10$ as such a choice works reasonably well for all the simulation experiments that we performed. Moreover, in our studies, we did not see any improvement in estimation accuracy for choices of m greater than 10 (note that larger m leads to slower computation).

B Some required preliminaries

B.1 Kullback-Leibler divergence

The Kullback-Leibler (KL) divergence between two probability measures P and Q is defined as $\mathbb{D}_{KL}(P \,\|\, Q) = \int \log\left(\frac{\mathrm{d}P}{\mathrm{d}Q}\right)$. Intuitively, if P is the true data generating distribution and we consider a model with distribution Q, then the KL divergence is the expected loss of information in using the distribution Q to model the true underlying distribution P. If P and Q are n-dimensional multivariate normal distributions with zero means and covariance matrices Σ_1 and Σ_2, respectively, then the KL divergence has a closed form,

$$\mathbb{D}_{KL}(P \,\|\, Q) = \frac{1}{2}\left[\mathrm{tr}(\Sigma_2^{-1}\Sigma_1) + \log|\Sigma_2| - \log|\Sigma_1| - n\right], \tag{27}$$

where tr() denotes the trace of a matrix and $|\cdot|$ denotes the determinant of a matrix.

B.2 Matrix-normal distribution

The matrix-normal distribution is a generalization of multivariate normal distribution to random matrices. Let $\boldsymbol{Y}$ be an $p \times n$ random matrix. The random matrix $\boldsymbol{Y}$ is said to be distributed according to the matrix-normal distribution if its probability density function is given by,

$$p(\boldsymbol{Y}) = \frac{\exp\left(-\frac{1}{2}\mathrm{tr}[\Sigma^{-1}(\boldsymbol{Y}-M)^{\top}\Lambda^{-1}(\boldsymbol{Y}-M)]\right)}{(2\pi)^{pn/2}|\Sigma|^{p/2}|\Lambda|^{n/2}}.$$

This is denoted as $\boldsymbol{Y} \sim \mathscr{MN}_{p,n}(M,\Lambda,\Sigma)$, where M is the $p \times n$ mean matrix, Λ is the $p \times p$ row covariance matrix, and Σ is the $n \times n$ column covariance matrix. The matrix-normal distribution is related to a multivariate normal distribution as,

$$\begin{aligned} &\boldsymbol{Y} \sim \mathscr{MN}_{p,n}(M,\Lambda,\Sigma) \\ \Leftrightarrow\ &\mathrm{vec}(\boldsymbol{Y}) \sim \mathscr{N}_{pn}(\mathrm{vec}(M), \Sigma \otimes \Lambda), \end{aligned} \tag{28}$$

where $\otimes$ denotes the Kronecker product and $\mathrm{vec}(\cdot)$ denotes the vectorization of a matrix.

C Some theoretical results

The next lemma generalizes equation (27) to the KL divergence between two matrix-normal distributions.

Lemma 1. *Consider two centered matrix-normal distributions $P : \mathscr{MN}_{p,n}(0,\Lambda_1,\Sigma_1)$ and $Q : \mathscr{MN}_{p,n}(0,\Lambda_2,\Sigma_2)$. The KL divergence between P and Q is given by,*

$$\mathbb{D}_{KL}(P\,\|\,Q) = \frac{1}{2}\left[\mathrm{tr}\left((\Sigma_2^{-1}\Sigma_1)\otimes(\Lambda_2^{-1}\Lambda_1)\right) - p\log\frac{|\Sigma_1|}{|\Sigma_2|} - n\log\frac{|\Lambda_1|}{|\Lambda_2|} - pn\right]. \tag{29}$$

Proof. The matrix-normal distribution is related to a multivariate normal distribution as,

$$\boldsymbol{Y} \sim \mathscr{MN}_{p,n}(M,\Lambda,\Sigma) \Leftrightarrow \mathrm{vec}(\boldsymbol{Y}) \sim \mathscr{N}_{pn}(\mathrm{vec}(M),\Sigma\otimes\Lambda), \tag{30}$$

where $\otimes$ denotes the Kronecker product and $\mathrm{vec}(\cdot)$ denotes the vectorization of a matrix. Using this equivalent representation of matrix-normal distribution and from the KL divergence between two multivariate normal distributions, we have,

$$\begin{aligned} \mathbb{D}_{KL}(P\,\|\,Q) &= \frac{1}{2}\left[\mathrm{tr}\left((\Sigma_2^{-1}\Sigma_1)\otimes(\Lambda_2^{-1}\Lambda_1)\right) + \log|\Sigma_2\otimes\Lambda_2| - \log|\Sigma_1\otimes\Lambda_1| - pn\right] \\ &= \frac{1}{2}\left[\mathrm{tr}\left((\Sigma_2^{-1}\Sigma_1)\otimes(\Lambda_2^{-1}\Lambda_1)\right) + \log\left(|\Sigma_2|^p|\Lambda_2|^n\right) - \log\left(|\Sigma_1|^p|\Lambda_1|^n\right) - pn\right] \\ &= \frac{1}{2}\left[\mathrm{tr}\left((\Sigma_2^{-1}\Sigma_1)\otimes(\Lambda_2^{-1}\Lambda_1)\right) - p\log\frac{|\Sigma_1|}{|\Sigma_2|} - n\log\frac{|\Lambda_1|}{|\Lambda_2|} - pn\right] \end{aligned}$$

The second equality follows as $|A\otimes B| = (|A|^p|B|^q)$ for $A \in \mathbb{R}^{q\times q}, B \in \mathbb{R}^{p\times p}$, and the last equality follows from rearranging the terms. □

We next theoretically show that a method ignoring the row dependence is sub-optimal in the KL sense when the true distribution has row dependence.

Theorem 1. *Consider the centered matrix-normal distribution $P : \mathscr{MN}_{p,n}(0,\Lambda,\Sigma)$, where both the column and row covariance matrices are positive definitive. Let L be any $n \times n$ lower-triangular matrix. Denote by $Q : \mathscr{MN}_{p,n}\left(0,\mathbb{I}_p,\left(LL^{\top}\right)^{-1}\right)$ and $R : \mathscr{MN}_{p,n}\left(0,\Lambda,\left(LL^{\top}\right)^{-1}\right)$. Further if $\lambda_1 < \cdots < \lambda_p$ denote the eigenvalues of Λ, then there exists a λ^* and λ^{**} depending on L and Σ, such that for $\lambda_1 \geq \lambda^*$ or $\lambda_p \leq \lambda^{**}$,*

$$\mathbb{D}_{KL}(P\,\|\,Q) \geq \mathbb{D}_{KL}(P\,\|\,R), \tag{31}$$

with equality holding if $\Lambda = \mathbb{I}_p$.

Proof. From Lemma 1, we have

$$\begin{aligned}\mathbb{D}_{KL}(P\,\|\,Q) &= \frac{1}{2}\left[\operatorname{tr}\left(\left(LL^\top\Sigma\right)\otimes\Lambda\right) - p\log\frac{|\Sigma|}{|(LL^\top)^{-1}|} - n\log|\Lambda| - pn\right] \\ &= \frac{1}{2}\left[p\left\{\operatorname{tr}\left(LL^\top\Sigma\right) - \log\frac{|\Sigma|}{|(LL^\top)^{-1}|} - n\right\} + (\operatorname{tr}(\Lambda)-p)\operatorname{tr}\left(LL^\top\Sigma\right) - n\log|\Lambda|\right] \\ &= \mathbb{D}_{KL}(P\,\|\,R) + \frac{1}{2}\left[(\operatorname{tr}(\Lambda)-p)\operatorname{tr}\left(LL^\top\Sigma\right) - n\log|\Lambda|\right] \end{aligned} \tag{32}$$

Note that $\varepsilon = \operatorname{tr}\left(LL^\top\Sigma\right) > 0$, as Σ and $LL^\top$ are positive-definite. Since $0 < \lambda_1 < \cdots < \lambda_p$ are the eigenvalues of Λ,

$$(\operatorname{tr}(\Lambda)-p)\operatorname{tr}\left(LL^\top\Sigma\right) - n\log|\Lambda| = \sum_{i=1}^{p}\{(\lambda_i - 1)\varepsilon - n\log\lambda_i\} \tag{33}$$

It is easy to see that the function $f(\lambda) = (\lambda-1)\varepsilon - n\log\lambda$ is convex and is minimized at $\lambda = n/\varepsilon$. Further at the minimizer n/ε, $f(\lambda) = 0$ if and only if $n/\varepsilon = 1$. For $n/\varepsilon \neq 1$, $f(\lambda) < 0$ at the minimizer. Since f is convex, there exists a $\lambda^* \geq n/\varepsilon$, i.e., a λ^* depending on L and Σ such that for any $\lambda \geq \lambda^*$, $f(\lambda) \geq 0$. Similarly, there exists a $\lambda^{**} \leq n/\varepsilon$ depending on L and Σ such that for any $\lambda \leq \lambda^{**}$, $f(\lambda) \geq 0$. The result follows immediately from equation (33) and equation (32). □

Theorem 1 suggests that existing spatial methods such as [14], which ignore the row dependence (i.e., setting $\Lambda = \mathbb{I}_p$), may not perform well for spatial transcriptomic data where the rows (i.e., genes) are correlated.

D Simulations

In this section we provide simulations to demonstrate the performance of the proposed method in the estimation of row and column covariance matrices for a matrix-normal distribution. We compare our method with the existing Bayesian nonparametric method of spatial covariance estimation for multivariate data [14], which is a special case of our proposed method. We consider the case of a single sample of matrix-variate data in Section D.1 and the multi-sample case in Section D.2.

D.1 Single-sample case

We considered $n = 100, 200, 500$ spatial locations and $p = 20, 30$ genes. The outline of the data generation and simulation strategies are as follows:

- We drew n spatial locations from $\mathscr{U}(0,1)\times\mathscr{U}(0,1)$, where $\mathscr{U}(0,1)$ denotes a uniform distribution on the interval $(0,1)$.
- The true column covariance Σ was generated from a Matérn covariance kernel with smoothness parameter equal to 0.25, marginal variance equal to 1, and varied the range parameter ($\phi = 1,2$), using the n random spatial locations generated.
- The true row covariance Λ was generated from $\mathscr{IW}(p,\Psi)$ where the scale matrix Ψ was considered to be one of the following choices:

AR-correlation	Equi-correlation	Banded-correlation
$\Psi_{ij} = \rho^{\lvert i-j\rvert}$	$\Psi_{ij} = \rho^{\mathbb{1}(i\neq j)}$	$\Psi_{ij} = \rho^{1-\mathbb{1}(i=j)}\mathbb{1}(\lvert i-j\rvert \in \{0,1\})$

Table S1. Choices of scale matrix Ψ for generating the true Λ. For all cases, $\rho = 0.5$.

- We fixed $m = 10$ for all our simulations.
- We used an *adaptive-MCMC* algorithm for sampling the hyperparameters $\boldsymbol{\theta} = (\theta_1,\theta_2,\theta_3)^\top$, using the publicly available R package `adaptMCMC`. We initialized $(\theta_1,\theta_2,\theta_3) = (1,-1,0)$. The initial scale (shape) matrix required for the proposal distribution of the *adaptive-MCMC* algorithm was taken as $\begin{bmatrix} 0.05 & -0.04 & 0 \\ -0.04 & 0.05 & 0 \\ 0 & 0 & 0.01 \end{bmatrix}$
- We ran $1,000$ iterations of our sampler and discarded the first $5,00$ samples as burn-in.
- To monitor convergence of our MCMC, we plotted the traceplots of log likelihood.

- Since for the matrix-normal distribution, the covariance matrices are non-identifiable, we consider the posterior correlation matrices and carry out comparisons based on the correlation matrix rather than the covariance matrix. We still denote by Σ, Λ, etc. the correlation matrices rather than the covariance matrix.
- We performed our simulations for 30 independent replicates for any given combination of simulation parameters.

We compared $\text{KL}_{\mathscr{N}}$ and $\text{KL}_{\mathscr{M}\mathscr{N}}$, which denote the KL divergence (in log scale) by assuming that $\boldsymbol{Y} \sim \mathscr{M}\mathscr{N}_{p,n}(0, \mathbb{I}, \Sigma)$ and $\boldsymbol{Y} \sim \mathscr{M}\mathscr{N}_{p,n}(0, \Lambda, \Sigma)$, respectively. Note that $\mathscr{M}\mathscr{N}_{p,n}(0, \mathbb{I}, \Sigma)$ corresponds to p independent realizations of a multivariate normal $\mathscr{N}_n(0, \Sigma)$. We compared both these quantities from our posterior estimates of Σ and/or Λ, denoting them with the subscript "P" and the MAP estimate of Σ obtained from the method by [14], denoting them with subscript "M". Hereafter, we refer to the method by [14] as the NPVecchia method and the corresponding estimates as NPVecchia estimates. We define the relative Frobenius error of an estimator $\hat{A}$ of the matrix A by,

$$RE_{\hat{A}} = \frac{\|\hat{A} - A\|_F}{\|A\|_F},$$

where F denotes the Frobenius norm of a matrix. We denote by RE_{Σ_P}, RE_{Λ_P}, and RE_{Σ_M} the relative Frobenius error of the correlation matrix Σ and Λ, with the subscript denoting the method used. Note that Λ is not estimated under NPVecchia method. Supplementary Table S2 summarizes the results of our simulations wherein we report the mean and standard deviation over the replicates for the different comparison metrics. It is clear that in situations when the rows of a matrix-variate data (genes) are correlated, the KL divergences and relative Frobenius errors are lower for our proposed method than that under the NPVecchia method. We also note that as the number of spatial locations increases, the accuracy of estimation of the row correlations increases as can be seen from the corresponding decreasing relative Frobenius error.

D.2 Multi-sample case

We further conducted our simulations for multiple independent samples. In particular we considered 3 independent samples, i.e. $R = 3$ of spatial data on the same set of genes (p) over possibly different spatial locations. For simplicity, we considered that the three samples have the same number of spatial locations i.e., $n_1 = n_2 = n_3 = n$. As before, we considered $n = 100, 200, 500$, and $p = 20, 30$. The outline of the data generation and simulation strategies are as follows:

- We drew $n_r,\ r = 1, 2, 3$ spatial locations independently from $\mathscr{U}(0,1) \times \mathscr{U}(0,1)$.
- The true column covariance Σ_1 for the sample 1 was generated from a Matérn covariance kernel with smoothness parameter equal to 0.5, marginal variance equal to 1, and range parameter equal to 2, using the n_1 random spatial locations generated.
- The true column covariance Σ_2 for the sample 2 was generated from a Matérn covariance kernel with smoothness parameter equal to 0.5, marginal variance equal to 1.5, and range parameter equal to 2, using the n_2 random spatial locations generated.
- The true column covariance Σ_3 for the sample 3 was generated from a Matérn covariance kernel with smoothness parameter equal to 0.5, marginal variance equal to 2, and range parameter equal to 2, using the n_3 random spatial locations generated.
- The true row covariance was generated from $\mathscr{I}\mathscr{W}(p, \Psi)$, where the scale matrix Ψ was considered to be one of the choices given in Supplementary Table S1.
- We performed our simulations for 30 independent replicates for any given combination of simulation parameters.
- All other data generation strategies, sampling parameters, and convergence criterion were similar to the single-sample case.

We calculated the relative Frobenius errors of the three spatial column correlation matrices and the row correlation matrix. From Supplementary Table S3, it can be seen that the relative Frobenius error from our posterior estimates of spatial correlation matrices are smaller than that from the NPVecchia estimates. Moreover, the relative Frobenius error from our posterior estimates decreases as p increases under any row correlation structure, whereas it shows an increasing trend for the NPVecchia estimates. Besides, as the number of spatial locations increases, the relative Frobenius error of the estimated row correlations decreases for the proposed method.

D.3 Scalability analysis

We looked at the empirical runtime of JOBS for increasing number of cells with fixed number of features (genes) using simulations. In particular, we first varied the number of cells from 500 to 3,000 with a fixed number $p = 30$ of genes. We ran 1,000 iterations of our sampler and reported the mean runtime along with the standard deviation over 30 independent replications. Supplementary Table S4 shows that JOBS scales nearly linearly with the number of cells. Second, we varied the number of features/genes from 20 to 150 with a fixed number $n = 1000$ of cells. Supplementary Table S5 shows that the runtime is sub-linear with the number of genes.

E Sensitivity analysis

In this section we provide simulations to demonstrate the performance of our proposed method in the estimation of row and column covariance matrices, when the data is in fact not distributed as matrix-normal. In particular, we considered the situation when the data is drawn from a matrix-variate t distribution.

E.1 Single-sample case

We considered $n = 100, 200, 500$ spatial locations and $p = 20$ genes. We also considered varying degrees of freedom of the corresponding matrix-variate t distribution The outline of the data generation and simulation strategies are as follows:

- We drew n spatial locations from $\mathscr{U}(0,1) \times \mathscr{U}(0,1)$, where $\mathscr{U}(0,1)$ denotes a uniform distribution on the interval $(0,1)$.
- The true column covariance Σ was generated from a Matérn covariance kernel with smoothness parameter equal to 0.25, marginal variance equal to 1, and the range parameter equal to 2, using the n random spatial locations generated.
- The true row covariance Λ was generated from $\mathscr{IW}(p, \Psi)$ where the scale matrix Ψ was considered to be one of the following choices:

AR-correlation	Equi-correlation	Banded-correlation
$\Psi_{ij} = \rho^{\lvert i-j \rvert}$	$\Psi_{ij} = \rho^{\mathbb{1}(i \neq j)}$	$\Psi_{ij} = \rho^{1-\mathbb{1}(i=j)} \mathbb{1}(\lvert i-j \rvert \in \{0,1\})$

Table S6. Choices of scale matrix Ψ for generating the true Λ. For all cases, $\rho = 0.5$.

- We fixed $m = 10$ for all our simulations.
- We used an *adaptive-MCMC* algorithm for sampling the hyperparameters $\boldsymbol{\theta} = (\theta_1, \theta_2, \theta_3)^\top$, using the publicly available R package `adaptMCMC`. We initialized $(\theta_1, \theta_2, \theta_3) = (1, -1, 0)$. The initial scale (shape) matrix required for the proposal distribution of the *adaptive-MCMC* algorithm was taken as $\begin{bmatrix} 0.05 & -0.04 & 0 \\ -0.04 & 0.05 & 0 \\ 0 & 0 & 0.01 \end{bmatrix}$
- We ran $1,000$ iterations of our sampler and discarded the first $5,00$ samples as burn-in.
- As in the matrix-normal distribution, the covariance matrices are non-identifiable, we consider the posterior correlation matrices and carry out comparisons based on the correlation matrix rather than the covariance matrix. We still denote by Σ, Λ, etc. the correlation matrices rather than the covariance matrix.
- We performed our simulations for 30 independent replicates for any given combination of simulation parameters.
- We calculated the relative Frobenius errors of the spatial column correlation matrix and the row correlation matrix to understand the estimation performance under this mis-specified model. We also looked at the relative Frobenius error of the spatial correlation matrix using the NP Vecchia method (denoted by RE_{Σ_M}).

Supplementary Table S7 shows that the estimation performance under our proposed method performs better than that obtained from the NPVecchia method as indicated by lower Relative Frobenius errors. It is worthwhile to note that under the mis-specified model, the estimation performance is sub-par in comparison to the case when the underlying data generating model is indeed matrix-variate Normal (see Supplementary Table S2). Also, an increase in the degrees of freedom of the matrix-variate t distribution shows an improved estimation of the row covariance matrix. However, the estimation of the column covariance matrix shows no such pattern. In the next section, we look at the estimation performance under the mis-specified model in the presence of multiple samples of the matrix-variate data.

E.2 Multi-sample case

We further conducted our sensitivity analysis for multiple independent samples from a matrix t-distribution. In particular we considered 3 and 5 independent samples, i.e. $R = 3,5$ of spatial data on the same set of genes (p) over possibly different spatial locations. For simplicity, we considered that the samples have the same number of spatial locations i.e., $n_r = n$, for all r. We considered the number of spatial locations, $n = 100,200,500$, and $p = 20$. The outline of the data generation and simulation strategies are as follows:

- We drew n_r spatial locations independently from $\mathscr{U}(0,1) \times \mathscr{U}(0,1)$.
- For the case 3-sample case, the true column covariance matrices were generated from a Matérn covariance kernel with smoothness parameter equal to 0.5, range parameter equal to 2, and the marginal variances equal to $1, 1.5$, and 2 respectively using the corresponding random spatial locations generated.
- For, the case 5-sample case, the first three true column covariance matrices were generated as before. The true column covariance matrices Σ_4 and Σ_5 for the samples 4 and 5 were generated from a Matérn covariance kernel with smoothness parameters equal to 0.5, range parameters equal to 1, and the marginal variances equal to 2 and 1 respectively, using the corresponding random spatial locations generated.
- The true row covariance was generated from $\mathscr{IW}(p,\Psi)$, where the scale matrix Ψ was considered to be of the AR-correlation type, as given in Supplementary Table S1.
- We generated the matrix variate data from a matrix t-distribution, with varying the degrees of freedom.
- We performed our simulations for 30 independent replicates for any given combination of simulation parameters.
- All other data generation strategies, sampling parameters, and convergence criterion were similar to the single-sample case.
- We calculated the relative Frobenius errors of the spatial column correlation matrices and the row correlation matrix to understand the estimation performance under this mis-specified model.

Clearly, as the number of replicates increase the Relative Frobenius errors of posterior row correlation matrices decreases under mis-specified model. The spatial correlation matrices show lower Relative Frobenius errors in comparison to the single-sample case. This provides evidence of improved estimation performance under the mis-specified model, highlighting the importance of multiple samples of spatial transcriptomic data.

F Real Data Analysis

For the STARmap data with two light samples, we ran the proposed JOBS. More concretely, we ran the Metropolis-Within-Blocked Gibbs sampler underlying JOBS for 1,500 iterations and considered a burn-in of 500 samples. The traceplot of log-likelihood showed no lack of convergence (Supplementary Fig. S1a) and the corresponding ACF plots in Supplementary Fig. S1b showed no significant correlation and hence no thinning of the samples were considered.
Furthermore, let us partition $\boldsymbol{Y} = (\boldsymbol{Y}_1\ \boldsymbol{Y}_2)$, where $\boldsymbol{Y}_1 \in \mathbb{R}^{p\times n-1}$ and $\boldsymbol{Y}_2 \in \mathbb{R}^{p\times 1}$. Accordingly, we partition Σ as,

$$\overset{n\times n}{\Sigma} = \begin{pmatrix} \overset{n-1\times n-1}{\Sigma_{11}} & \overset{n-1\times 1}{\Sigma_{12}} \\ \overset{1\times n-1}{\Sigma_{21}} & \overset{1\times 1}{\Sigma_{22}} \end{pmatrix}.$$

From standard results on matrix normal distribution, we have

$$\boldsymbol{Y}_2 \mid \boldsymbol{Y}_1 \sim \mathscr{N}_p\left(\boldsymbol{Y}_1\Sigma_{11}^{-1}\Sigma_{12},\ \Lambda \otimes \left(\Sigma_{22} - \Sigma_{21}\Sigma_{11}^{-1}\Sigma_{12}\right)\right).$$

Note that $\boldsymbol{Y}_2$ corresponds to the gene expression data measured on the set of all p genes and the single cell n. The corresponding fitted gene expression is given by $\boldsymbol{Y}_1\hat{\Sigma}_{11}^{-1}\hat{\Sigma}_{12}$, where $\hat{\Sigma}$ is the posterior estimate of the spatial correlation matrix obtained from JOBS with $\hat{\Sigma}_{11}$ and $\hat{\Sigma}_{12}$ denoting the corresponding estimated sub-matrices. We can progressively reorder the data such that each cell corresponds to the last column of the reordered data $\boldsymbol{Y}$, i.e., $\boldsymbol{Y}_2$ in the corresponding partition. For each such cell, we can obtain the fitted gene expression data using the posterior estimate of the spatial correlation matrix. This gives the fitted gene expression data for the set of all p genes and n single cells obtained from JOBS. These fitted gene expression data may be considered as a "de-noised" or smooth representation of the raw gene expression data and may be used for further downstream analyses.

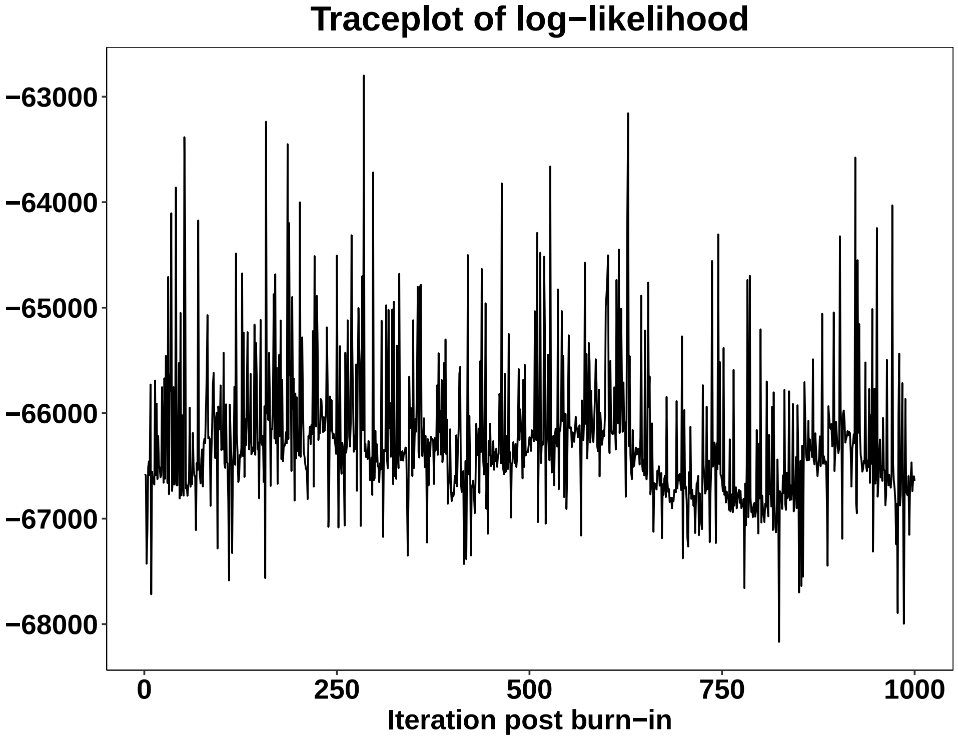

(a) Traceplot of log-likelihood

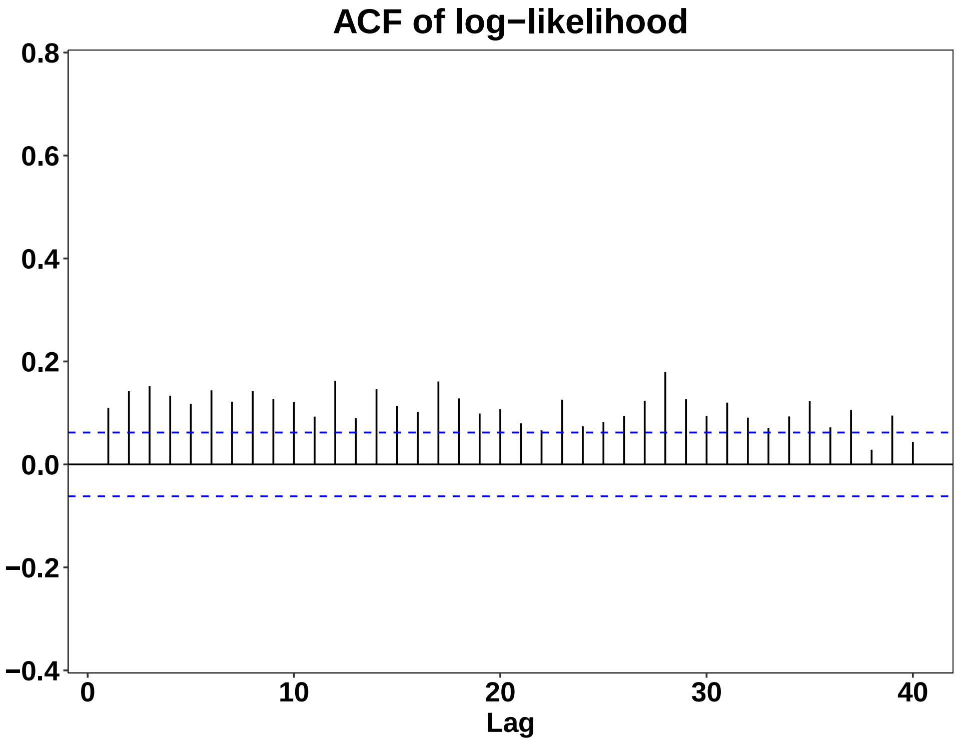

(b) ACF Plot of log-likelihood.

Figure S1. The traceplot and ACF of log-likelihood post burn-in and thinning for JOBS.

We considered a Gaussian mixture model (GMM) on the smoothed gene expression data corresponding to each sample for spatial clustering of the cells and chose the optimal number of clusters using the Bayesian Information Criterion [15]. In the

main manuscript we presented the clustering plot from JOBS, *DR.SC* [16], and *BayesSpace* [17] for one light sample. Here we present the clustering plot for the other light sample in Supplementary Fig. S2. As before, since *BayesSpace* requires the specification of the number of clusters, we ran the algorithm for multiple choices of the number of clusters and report the one with highest accuracy.

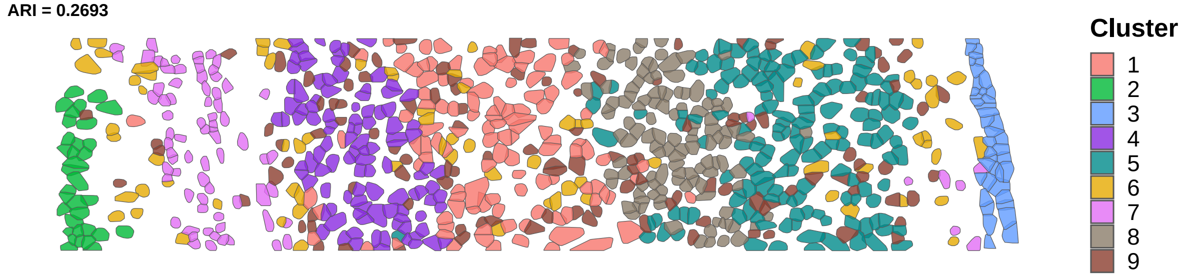

(a) JOBS.

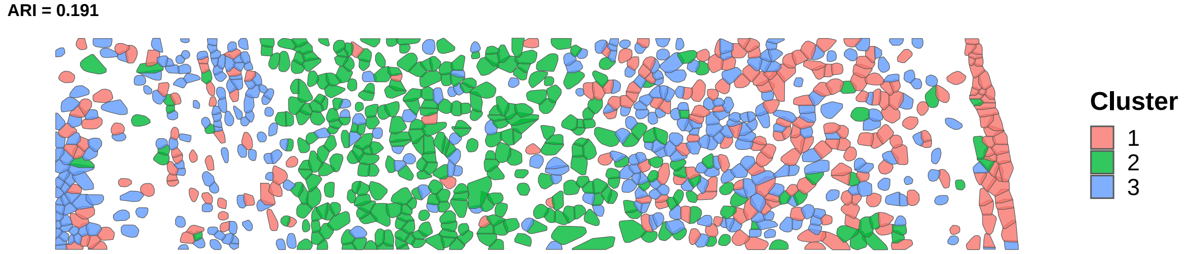

(b) `DR.SC`.

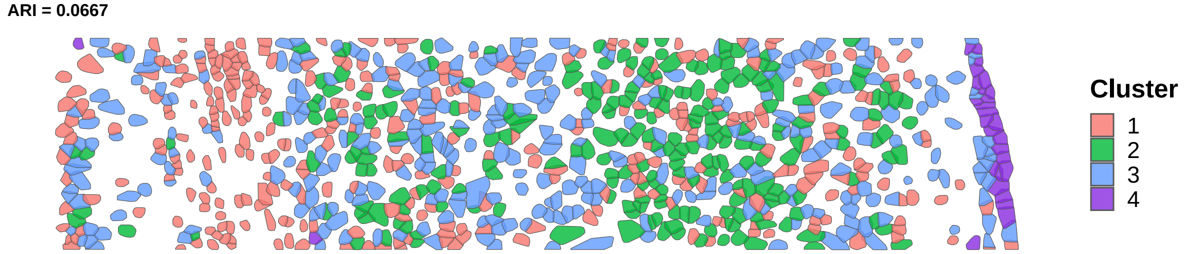

(c) BayesSpace.

Figure S2. Spatial clustering from our proposed JOBS compared with the state of the art method as implemented by `DR.SC` R package and the BayesSpace for one of the light samples. The colors indicate the estimated clusters. ARI comparing the estimated cluster labels with the manually annotated cells is reported at the top of each panel.

We looked at the Adjusted Rand Index [18] to demonstrate clustering performance of the three competing methods,

comparing the estimated cluster labels with the manually annotated cell types of excitatory cells. Clearly, the clustering obtained from JOBS outperformed the other two methods in terms of clustering accuracy.

Next, we have applied JOBS on another dataset obtained by [19] using the 10x Genomics Visium technology. The dataset consisted of the gene expression of 33,538 genes measured for 4,634 spots along with the spatial location of the spots for the six-layered dorsolateral prefrontal cortex (DLPFC) of the adult human brain. We also obtained the manually annotated Visium spots to objectively assess clustering performance. We selected the top 30 spatially varying genes and performed standard pre-processing of the data [20]. We ran JOBS on the processed dataset to jointly estimate the spatial and gene correlations. The mean correlation and the mean squared error between the smoothed and observed gene expression values were found to be 0.92 and 0.51 respectively. This indicated the high accuracy of the estimation of the gene and spatial-spot covariance matrices. The Supplementary Fig. S3 shows that smoothed and observed spatial expression patterns for the two genes are highly aligned. Furthermore, using the smoothed gene expression data, we considered spatial clustering of the spots. We compared the clustering performance of our method with that obtained from *DR.SC* and *BayesSpace*. In all methods, we fixed the number of clusters to be equal to the true number of clusters obtained from the manually annotated labels to ensure a fair comparison. The Supplementary Fig. S4 shows the corresponding clustering plots. Clearly, the JOBS-based clustering outperformed the other methods.

Additionally, we considered a human breast cancer dataset obtained from 10x Visium platform as studied by [21]. Particularly, the dataset consisted of the gene expression data from 2,518 spots and 17,651 genes measured on a section of human breast with invasive ductal carcinoma, along with the manual annotation from pathologists. We selected the top 50 spatially varying genes and ran JOBS to jointly estimate the spatial and gene correlations. As before, we compared the clustering performance of our JOBS smoothed data with that of *DR.SC* and *BayesSpace*. We see from Supplementary Fig. S5 that JOBS-based clustering performance is comparable (slightly better) to the other two methods.

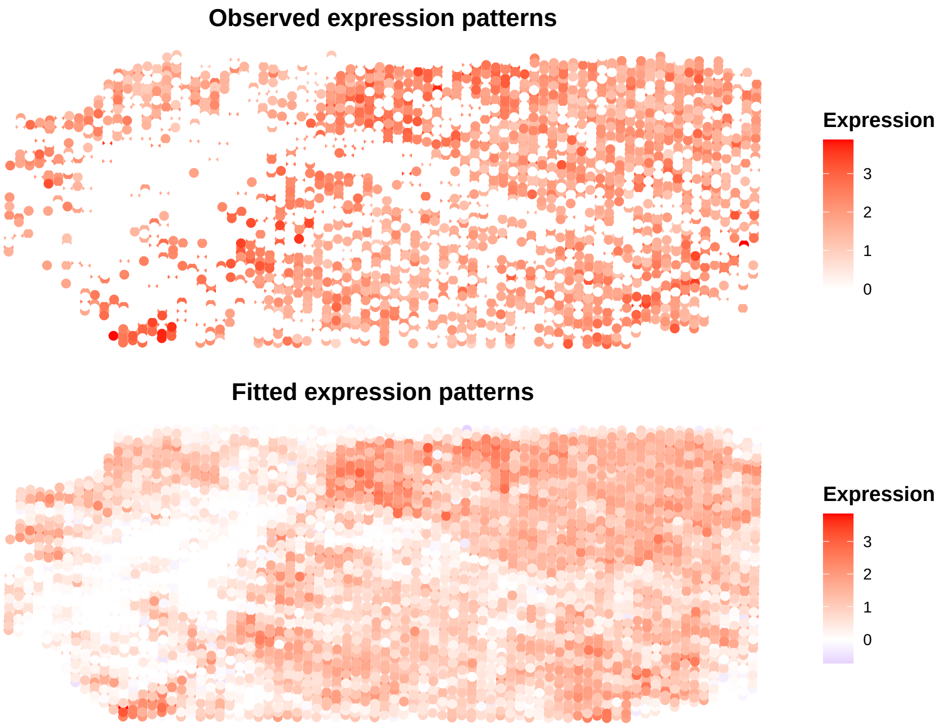

(a) Gene **ENSG00000185499**.

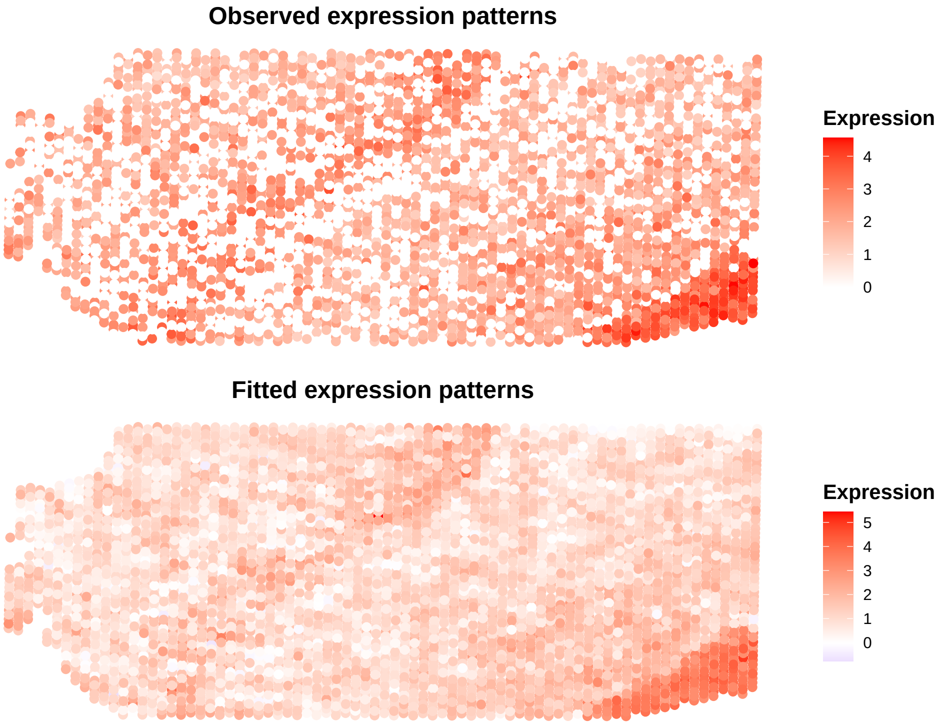

(b) Gene **ENSG00000109846**.

Figure S3. Observed and smoothed spatial expression patterns for two genes corresponding to the DLPFC dataset.

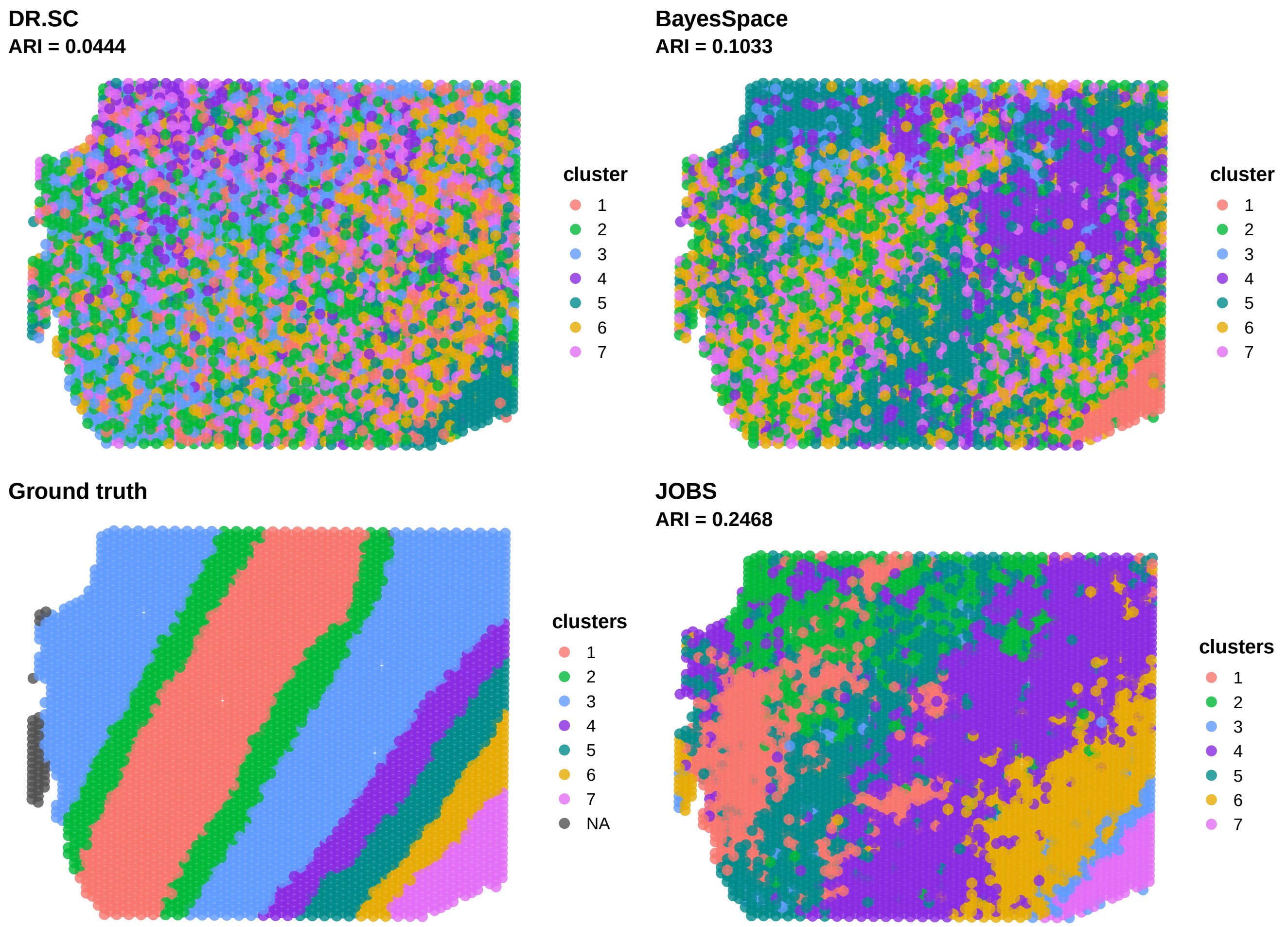

Figure S4. Spatial clustering using *DR.SC*, *BayesSpace*, JOBS, and the true cluster labels. The Adjusted Rand Index comparing the clustering performance with the true clusters are reported at the top of each panel.

ϕ	p	n	Correlation	$KL_{\mathcal{N}(P)}$	$KL_{\mathcal{N}(M)}$	$KL_{\mathcal{MN}(P)}$	$KL_{\mathcal{MN}(M)}$	RE_{Σ_P}	RE_{Λ_P}	RE_{Σ_M}
1	20	100	AR	2.465 (0.290)	6.505 (0.447)	6.126 (0.245)	9.608 (0.407)	0.474 (0.138)	0.252 (0.071)	0.976 (0.135)
		200	AR	2.989 (0.110)	7.242 (0.295)	6.404 (0.112)	10.337 (0.270)	0.501 (0.115)	0.178 (0.051)	0.991 (0.153)
		500	AR	3.883 (0.097)	8.462 (0.358)	7.089 (0.087)	11.533 (0.332)	0.465 (0.129)	0.115 (0.031)	1.033 (0.160)
		100	Equi	2.465 (0.268)	6.501 (0.386)	6.078 (0.225)	9.603 (0.347)	0.456 (0.141)	0.246 (0.065)	0.944 (0.152)
		200	Equi	2.922 (0.114)	7.099 (0.410)	6.333 (0.132)	10.211 (0.372)	0.465 (0.126)	0.164 (0.038)	0.943 (0.122)
		500	Equi	3.868 (0.100)	8.364 (0.388)	7.079 (0.116)	11.443 (0.359)	0.443 (0.133)	0.105 (0.028)	0.963 (0.133)
		100	Banded	2.400 (0.182)	7.182 (0.339)	6.309 (0.136)	10.245 (0.319)	0.513 (0.130)	0.234 (0.046)	1.017 (0.166)
		200	Banded	2.959 (0.134)	8.122 (0.308)	6.666 (0.157)	11.170 (0.293)	0.497 (0.136)	0.167 (0.033)	1.044 (0.138)
		500	Banded	3.898 (0.143)	9.491 (0.311)	7.317 (0.144)	12.52 (0.301)	0.434 (0.139)	0.106 (0.02)	1.059 (0.121)
	30	100	AR	2.227 (0.241)	8.745 (0.440)	6.657 (0.207)	12.174 (0.431)	0.441 (0.122)	0.124 (0.040)	1.38 (0.185)
		200	AR	2.754 (0.152)	9.634 (0.309)	6.938 (0.183)	13.057 (0.303)	0.366 (0.119)	0.088 (0.019)	1.457 (0.175)
		500	AR	3.650 (0.134)	10.611 (0.239)	7.474 (0.165)	14.033 (0.235)	0.353 (0.120)	0.052 (0.012)	1.434 (0.182)
		100	Equi	2.237 (0.333)	8.617 (0.332)	6.655 (0.314)	12.048 (0.322)	0.445 (0.124)	0.136 (0.041)	1.337 (0.176)
		200	Equi	2.78 (0.210)	9.531 (0.37)	6.954 (0.218)	12.957 (0.362)	0.41 (0.125)	0.089 (0.027)	1.394 (0.172)
		500	Equi	3.576 (0.073)	10.451 (0.343)	7.417 (0.134)	13.876 (0.335)	0.389 (0.086)	0.056 (0.015)	1.448 (0.159)
		100	Banded	2.316 (0.294)	9.051 (0.401)	7.037 (0.285)	12.474 (0.393)	0.409 (0.09)	0.124 (0.044)	1.376 (0.158)
		200	Banded	2.757 (0.141)	10.087 (0.305)	7.322 (0.258)	13.503 (0.3)	0.397 (0.124)	0.09 (0.025)	1.394 (0.161)
		500	Banded	3.584 (0.083)	11.34 (0.352)	7.724 (0.214)	14.752 (0.348)	0.387 (0.108)	0.052 (0.015)	1.521 (0.187)
2	20	100	AR	2.411 (0.161)	6.283 (0.405)	6.025 (0.177)	9.409 (0.355)	0.416 (0.181)	0.258 (0.059)	0.664 (0.160)
		200	AR	3.016 (0.209)	7.011 (0.422)	6.416 (0.197)	10.134 (0.375)	0.387 (0.158)	0.179 (0.041)	0.643 (0.142)
		500	AR	3.865 (0.105)	8.240 (0.394)	7.096 (0.119)	11.329 (0.36)	0.390 (0.129)	0.134 (0.036)	0.611 (0.105)
		100	Equi	2.379 (0.204)	6.194 (0.530)	5.957 (0.170)	9.338 (0.457)	0.453 (0.183)	0.271 (0.068)	0.618 (0.164)
		200	Equi	2.958 (0.152)	7.141 (0.611)	6.400 (0.139)	10.255 (0.563)	0.416 (0.148)	0.196 (0.061)	0.608 (0.223)
		500	Equi	3.842 (0.143)	8.021 (0.329)	7.055 (0.125)	11.130 (0.295)	0.431 (0.138)	0.127 (0.035)	0.594 (0.158)
		100	Banded	2.467 (0.265)	6.987 (0.619)	6.470 (0.225)	10.072 (0.575)	0.389 (0.154)	0.286 (0.061)	0.639 (0.137)
		200	Banded	3.058 (0.232)	8.201 (0.461)	6.819 (0.276)	11.248 (0.441)	0.403 (0.195)	0.202 (0.057)	0.637 (0.167)
		500	Banded	3.843 (0.120)	9.346 (0.616)	7.307 (0.145)	12.384 (0.600)	0.355 (0.123)	0.111 (0.034)	0.712 (0.181)
	30	100	AR	2.322 (0.358)	8.516 (0.523)	6.692 (0.294)	11.952 (0.511)	0.375 (0.157)	0.134 (0.039)	1.020 (0.219)
		200	AR	2.738 (0.128)	9.541 (0.545)	6.933 (0.203)	12.968 (0.534)	0.349 (0.143)	0.090 (0.022)	1.045 (0.228)
		500	AR	3.598 (0.136)	10.578 (0.416)	7.484 (0.170)	14.001 (0.408)	0.312 (0.111)	0.068 (0.024)	0.945 (0.227)
		100	Equi	2.247 (0.253)	8.616 (0.416)	6.677 (0.281)	12.048 (0.403)	0.454 (0.162)	0.153 (0.047)	0.943 (0.217)
		200	Equi	2.801 (0.185)	9.231 (0.319)	6.918 (0.192)	12.664 (0.309)	0.370 (0.168)	0.097 (0.032)	0.992 (0.211)
		500	Equi	3.638 (0.156)	10.545 (0.490)	7.454 (0.145)	13.969 (0.482)	0.379 (0.129)	0.059 (0.020)	0.958 (0.236)
		100	Banded	2.232 (0.211)	9.005 (0.518)	7.049 (0.288)	12.43 (0.508)	0.359 (0.146)	0.142 (0.047)	0.970 (0.204)
		200	Banded	2.843 (0.197)	9.976 (0.459)	7.286 (0.131)	13.395 (0.452)	0.310 (0.128)	0.093 (0.022)	0.988 (0.206)
		500	Banded	3.635 (0.123)	11.088 (0.431)	7.836 (0.147)	14.504 (0.425)	0.341 (0.118)	0.056 (0.014)	1.007 (0.166)

Table S2. KL divergences (in log scale) and Relative Frobenius error for the two methods, with varying number of genes, number of spatial locations, and range of Mateŕn kernel using estimated correlation matrices for the different choices of the scale matrix for the true row correlation (lower values indicate better fit). We report the mean (s.d) over 30 independent replicates.

p	n_r	Correlation	$RE_{\Sigma_{1(\mathrm{P})}}$	$RE_{\Sigma_{2(\mathrm{P})}}$	$RE_{\Sigma_{3(\mathrm{P})}}$	$RE_{\Lambda_{\mathrm{P}}}$	$RE_{\Sigma_{1(\mathrm{M})}}$	$RE_{\Sigma_{2(\mathrm{M})}}$	$RE_{\Sigma_{3(\mathrm{M})}}$
20	100	AR	0.093 (0.056)	0.087 (0.034)	0.100 (0.036)	0.130 (0.022)	0.333 (0.189)	0.304 (0.156)	0.320 (0.148)
	200	AR	0.102 (0.051)	0.090 (0.031)	0.087 (0.040)	0.090 (0.014)	0.260 (0.101)	0.245 (0.070)	0.313 (0.155)
	500	AR	0.079 (0.041)	0.072 (0.014)	0.093 (0.034)	0.059 (0.009)	0.279 (0.121)	0.287 (0.136)	0.275 (0.153)
	100	Equi	0.086 (0.035)	0.080 (0.030)	0.091 (0.038)	0.126 (0.021)	0.233 (0.086)	0.240 (0.119)	0.304 (0.167)
	200	Equi	0.076 (0.026)	0.085 (0.024)	0.090 (0.033)	0.089 (0.018)	0.255 (0.096)	0.238 (0.135)	0.295 (0.145)
	500	Equi	0.080 (0.026)	0.082 (0.026)	0.081 (0.027)	0.059 (0.010)	0.250 (0.115)	0.239 (0.100)	0.258 (0.133)
	100	Banded	0.096 (0.053)	0.081 (0.029)	0.094 (0.031)	0.126 (0.025)	0.308 (0.157)	0.295 (0.149)	0.283 (0.149)
	200	Banded	0.087 (0.041)	0.084 (0.029)	0.089 (0.025)	0.088 (0.017)	0.285 (0.130)	0.266 (0.121)	0.260 (0.120)
	500	Banded	0.106 (0.063)	0.073 (0.026)	0.095 (0.040)	0.062 (0.012)	0.329 (0.165)	0.251 (0.081)	0.271 (0.142)
30	100	AR	0.071 (0.025)	0.074 (0.022)	0.076 (0.030)	0.063 (0.016)	0.488 (0.283)	0.370 (0.171)	0.526 (0.289)
	200	AR	0.065 (0.017)	0.081 (0.027)	0.083 (0.026)	0.044 (0.012)	0.538 (0.267)	0.493 (0.237)	0.445 (0.217)
	500	AR	0.074 (0.029)	0.066 (0.021)	0.067 (0.026)	0.029 (0.008)	0.514 (0.249)	0.493 (0.262)	0.456 (0.226)
	100	Equi	0.073 (0.025)	0.069 (0.020)	0.077 (0.031)	0.070 (0.018)	0.423 (0.189)	0.498 (0.264)	0.424 (0.217)
	200	Equi	0.065 (0.023)	0.077 (0.026)	0.082 (0.027)	0.047 (0.011)	0.424 (0.202)	0.454 (0.216)	0.474 (0.24)
	500	Equi	0.074 (0.026)	0.068 (0.02)	0.078 (0.028)	0.028 (0.008)	0.528 (0.277)	0.443 (0.243)	0.443 (0.228)
	100	Banded	0.073 (0.020)	0.075 (0.022)	0.080 (0.026)	0.068 (0.017)	0.432 (0.171)	0.487 (0.254)	0.483 (0.275)
	200	Banded	0.069 (0.022)	0.075 (0.025)	0.079 (0.028)	0.046 (0.012)	0.469 (0.221)	0.430 (0.203)	0.482 (0.208)
	500	Banded	0.066 (0.022)	0.076 (0.026)	0.075 (0.026)	0.029 (0.008)	0.439 (0.23)	0.444 (0.249)	0.469 (0.259)

Table S3. Relative Frobenius error for each spatial correlation matrix and the row correlation matrix norm for the two methods. Results are presented with varying number of genes, number of spatial locations, and different choices of the scale matrix for the true row correlation (lower values indicate better fit). Note that under NPVecchia method, the row correlation is not estimated. We report the mean (s.d) over 30 independent replicates.

n	500	1000	2000	3000
Time	0.929 (0.006)	1.876 (0.019)	3.898 (0.035)	5.391 (0.668)

Table S4. Runtime of JOBS (in hours) against the number of spatial locations n. The number of features p was fixed at 30. We report the mean time (s.d) over 30 independent replicates.

p	20	30	50	100	150
Time	1.813 (0.011)	1.876 (0.019)	2.027 (0.013)	2.697 (0.048)	5.378 (0.028)

Table S5. Runtime of JOBS (in hours) against the number of features p. The number of spatial locations n was fixed at 1000. We report the mean runtime (s.d) over 30 independent replicates.

df	n	Correlation	RE_{Σ_P}	RE_{Λ_P}	RE_{Σ_M}
3	100	AR	0.633 (0.112)	0.924 (0.241)	0.874 (0.278)
	200	AR	0.566 (0.096)	1.003 (0.327)	0.866 (0.274)
	500	AR	0.479 (0.127)	0.957 (0.220)	0.913 (0.222)
	100	Equi	0.631 (0.115)	1.023 (0.325)	0.946 (0.293)
	200	Equi	0.652 (0.117)	0.858 (0.240)	0.876 (0.243)
	500	Equi	0.574 (0.092)	0.948 (0.223)	0.913 (0.253)
	100	Banded	0.652 (0.146)	0.885 (0.204)	0.921 (0.313)
	200	Banded	0.624 (0.103)	0.922 (0.287)	0.898 (0.229)
	500	Banded	0.558 (0.107)	0.867 (0.214)	0.885 (0.261)
5	100	AR	0.632 (0.118)	0.757 (0.175)	0.893 (0.308)
	200	AR	0.597 (0.125)	0.763 (0.156)	0.874 (0.220)
	500	AR	0.490 (0.137)	0.766 (0.222)	0.832 (0.287)
	100	Equi	0.691 (0.095)	0.802 (0.235)	0.920 (0.307)
	200	Equi	0.662 (0.109)	0.755 (0.186)	0.865 (0.263)
	500	Equi	0.545 (0.127)	0.753 (0.189)	0.827 (0.251)
	100	Banded	0.658 (0.109)	0.751 (0.193)	0.844 (0.225)
	200	Banded	0.647 (0.117)	0.751 (0.158)	0.795 (0.232)
	500	Banded	0.589 (0.139)	0.803 (0.254)	0.876 (0.223)

Table S7. Relative Frobenius error when the data is drawn from a matrix t-distribution, with varying degrees of freedom of the t-distribution, and number of spatial locations for the different choices of the scale matrix for the true row correlation (lower values indicate better fit). We report the mean (s.d) over 30 independent replicates.

R	df	n	$RE_{\Sigma_{1(P)}}$	$RE_{\Sigma_{2(P)}}$	$RE_{\Sigma_{3(P)}}$	$RE_{\Sigma_{4(P)}}$	$RE_{\Sigma_{5(P)}}$	RE_{Λ_P}
3	3	100	0.291 (0.191)	0.283 (0.216)	0.284 (0.160)			0.374 (0.078)
		200	0.191 (0.085)	0.296 (0.205)	0.266 (0.163)			0.393 (0.083)
		500	0.283 (0.174)	0.273 (0.158)	0.283 (0.131)			0.386 (0.071)
	5	100	0.183 (0.141)	0.180 (0.104)	0.250 (0.167)			0.354 (0.056)
		200	0.206 (0.145)	0.208 (0.139)	0.207 (0.152)			0.340 (0.058)
		500	0.181 (0.089)	0.176 (0.078)	0.243 (0.144)			0.356 (0.064)
5	3	100	0.259 (0.179)	0.238 (0.102)	0.326 (0.212)	0.517 (0.178)	0.546 (0.318)	0.272 (0.059)
		200	0.273 (0.147)	0.299 (0.204)	0.284 (0.157)	0.475 (0.164)	0.490 (0.211)	0.261 (0.035)
		500	0.257 (0.232)	0.245 (0.170)	0.230 (0.139)	0.558 (0.222)	0.581 (0.242)	0.255 (0.044)
	5	100	0.179 (0.122)	0.233 (0.166)	0.175 (0.092)	0.431 (0.143)	0.399 (0.153)	0.256 (0.053)
		200	0.211 (0.137)	0.212 (0.110)	0.230 (0.156)	0.389 (0.123)	0.370 (0.156)	0.257 (0.044)
		500	0.187 (0.118)	0.227 (0.127)	0.225 (0.129)	0.412 (0.118)	0.387 (0.194)	0.237 (0.031)

Table S8. Relative Frobenius error when the data is drawn from a matrix t-distribution, with varying degrees of freedom of the t-distribution, number of spatial locations, and different number of samples of the matrix-variate data (lower values indicate better fit). We report the mean (s.d) over 30 independent replicates.

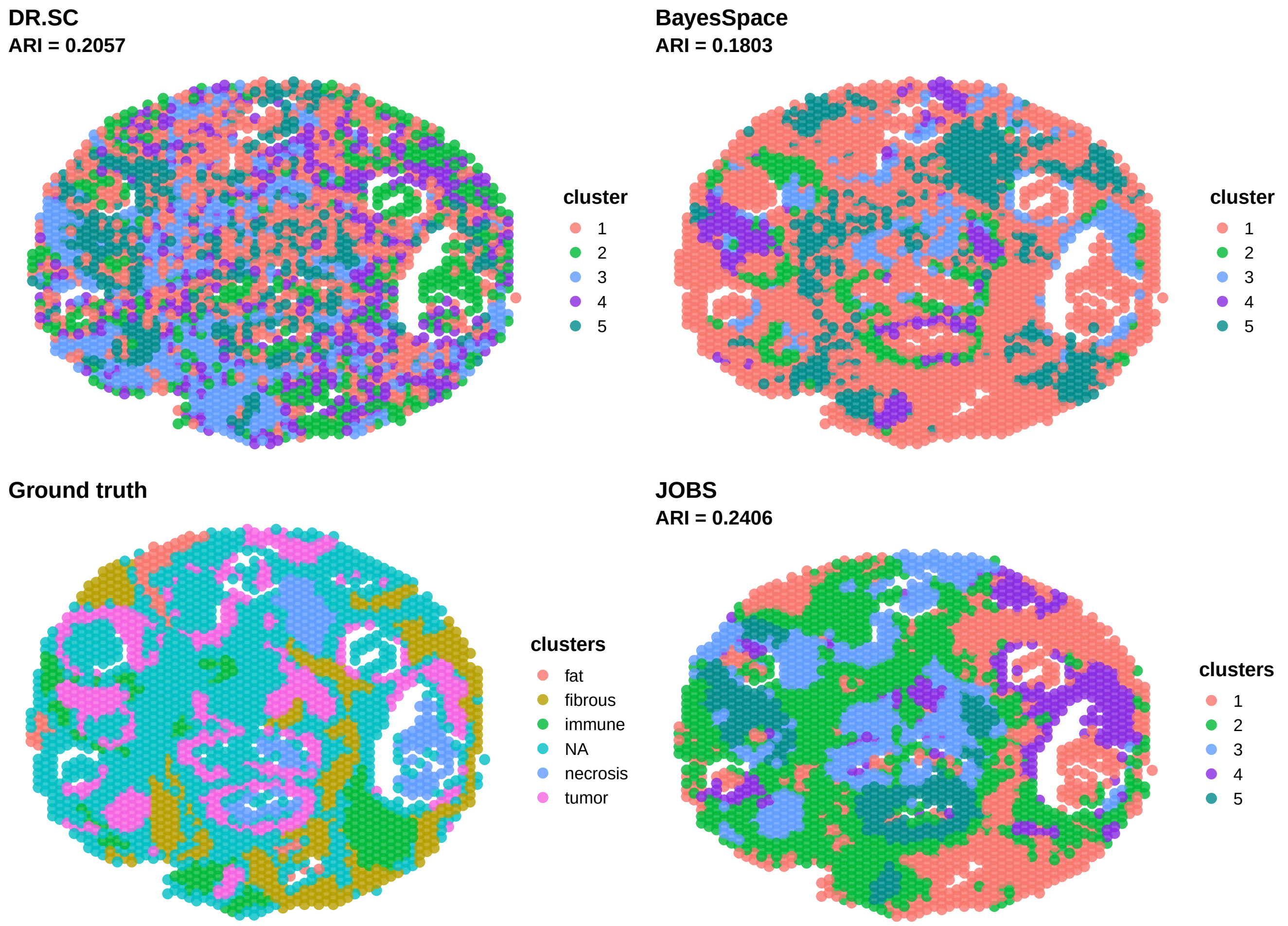

Figure S5. Spatial clustering using *DR.SC*, *BayesSpace*, JOBS, and the true manually annotated labels for the human breast cancer data. The Adjusted Rand Index comparing the clustering performance with the true clusters are reported at the top of each panel.

References

1. Pourahmadi, M. Cholesky Decompositions and Estimation of A Covariance Matrix: Orthogonality of Variance–Correlation Parameters. *Biometrika* **94**, 1006–1013, DOI: 10.1093/biomet/asm073 (2007). https://academic.oup.com/biomet/article-pdf/94/4/1006/681653/asm073.pdf.
2. Guinness, J. Permutation and grouping methods for sharpening gaussian process approximations. *Technometrics* **60**, 415–429, DOI: 10.1080/00401706.2018.1437476 (2018). PMID: 31447491, https://doi.org/10.1080/00401706.2018.1437476.
3. Schäfer, F., Sullivan, T. J. & Owhadi, H. Compression, inversion, and approximate pca of dense kernel matrices at near-linear computational complexity. *Multiscale Model. & Simul.* **19**, 688–730, DOI: 10.1137/19M129526X (2021). https://doi.org/10.1137/19M129526X.
4. Vecchia, A. V. Estimation and model identification for continuous spatial processes. *J. Royal Stat. Soc. Ser. B (Methodological)* **50**, 297–312 (1988).
5. Stein, M. L., Chi, Z. & Welty, L. J. Approximating likelihoods for large spatial data sets. *J. Royal Stat. Soc. Ser. B (Statistical Methodol.* **66**, 275–296, DOI: https://doi.org/10.1046/j.1369-7412.2003.05512.x (2004). https://rss.onlinelibrary.wiley.com/doi/pdf/10.1046/j.1369-7412.2003.05512.x.
6. Datta, A., Banerjee, S., Finley, A. O. & Gelfand, A. E. Hierarchical nearest-neighbor gaussian process models for large geostatistical datasets. *J. Am. Stat. Assoc.* **111**, 800–812, DOI: 10.1080/01621459.2015.1044091 (2016). PMID: 29720777, https://doi.org/10.1080/01621459.2015.1044091.
7. Katzfuss, M., Guinness, J., Gong, W. & Zilber, D. Vecchia approximations of gaussian-process predictions. *J. Agric. Biol. Environ. Stat.* **25**, 383–414, DOI: 10.1007/s13253-020-00401-7 (2020).
8. Katzfuss, M. & Guinness, J. A General Framework for Vecchia Approximations of Gaussian Processes. *Stat. Sci.* **36**, 124 – 141, DOI: 10.1214/19-STS755 (2021).
9. Schäfer, F., Katzfuss, M. & Owhadi, H. Sparse cholesky factorization by kullback–leibler minimization. *SIAM J. on Sci. Comput.* **43**, A2019–A2046, DOI: 10.1137/20M1336254 (2021). https://doi.org/10.1137/20M1336254.
10. Huang, J. Z., Liu, N., Pourahmadi, M. & Liu, L. Covariance matrix selection and estimation via penalised normal likelihood. *Biometrika* **93**, 85–98, DOI: 10.1093/biomet/93.1.85 (2006). https://academic.oup.com/biomet/article-pdf/93/1/85/645360/93185.pdf.
11. Bernardo, J. *et al.* Bayesian factor regression models in the "large p, small n" paradigm. *Bayesian statistics* **7**, 733–742 (2003).
12. Carvalho, C. M. *et al.* High-dimensional sparse factor modeling: Applications in gene expression genomics. *J. Am. Stat. Assoc.* **103**, 1438–1456, DOI: 10.1198/016214508000000869 (2008). PMID: 21218139, https://doi.org/10.1198/016214508000000869.
13. Bhattacharya, A. & Dunson, D. B. Sparse Bayesian infinite factor models. *Biometrika* **98**, 291–306, DOI: 10.1093/biomet/asr013 (2011). https://academic.oup.com/biomet/article-pdf/98/2/291/46695653/asr013.pdf.
14. Kidd, B. & Katzfuss, M. Bayesian Nonstationary and Nonparametric Covariance Estimation for Large Spatial Data (with Discussion). *Bayesian Analysis* **17**, 291 – 351, DOI: 10.1214/21-BA1273 (2022).
15. Neath, A. A. & Cavanaugh, J. E. The bayesian information criterion: background, derivation, and applications. *WIREs Comput. Stat.* **4**, 199–203, DOI: 10.1002/wics.199 (2012).
16. Liu, W. *et al.* Joint dimension reduction and clustering analysis of single-cell RNA-seq and spatial transcriptomics data. *Nucleic Acids Res.* **50**, e72–e72, DOI: 10.1093/nar/gkac219 (2022). https://academic.oup.com/nar/article-pdf/50/12/e72/44450490/gkac219.pdf.
17. Zhao, E. *et al.* Spatial transcriptomics at subspot resolution with bayesspace. *Nat. Biotechnol.* **39**, 1375–1384, DOI: 10.1038/s41587-021-00935-2 (2021).
18. Hubert, L. & Arabie, P. Comparing partitions. *J. Classif.* **2**, 193–218, DOI: 10.1007/BF01908075 (1985).
19. Maynard, K. R. *et al.* Transcriptome-scale spatial gene expression in the human dorsolateral prefrontal cortex. *Nat. neuroscience* **24**, 425–436 (2021).
20. Hao, Y. *et al.* Integrated analysis of multimodal single-cell data. *Cell* **184**, 3573–3587.e29, DOI: https://doi.org/10.1016/j.cell.2021.04.048 (2021).
21. Jiang, X. *et al.* Integrating image and molecular profiles for spatial transcriptomics analysis. *bioRxiv* DOI: 10.1101/2023.06.18.545488 (2023). https://www.biorxiv.org/content/early/2023/06/20/2023.06.18.545488.full.pdf.
